# Genome-wide identification of *Drosophila* dorso-ventral enhancers by differential histone acetylation analysis

**DOI:** 10.1186/s13059-016-1057-2

**Published:** 2016-09-27

**Authors:** Nina Koenecke, Jeff Johnston, Bjoern Gaertner, Malini Natarajan, Julia Zeitlinger

**Affiliations:** 1 Stowers Institute for Medical Research, Kansas City, MO 64110 USA; 2 Present address: Department of Pediatrics, University of California, San Diego, La Jolla, CA 92093 USA; 3 Department of Pathology and Laboratory Medicine, University of Kansas Medical Center, Kansas City, KS 66160 USA

**Keywords:** Enhancer identification, CBP, ATAC-seq, H3K27ac

## Abstract

**Background:**

*Drosophila* dorso-ventral (DV) patterning is one of the best-understood regulatory networks to date, and illustrates the fundamental role of enhancers in controlling patterning, cell fate specification, and morphogenesis during development. Histone acetylation such as H3K27ac is an excellent marker for active enhancers, but it is challenging to obtain precise locations for enhancers as the highest levels of this modification flank the enhancer regions. How to best identify tissue-specific enhancers in a developmental system de novo with a minimal set of data is still unclear.

**Results:**

Using DV patterning as a test system, we develop a simple and effective method to identify tissue-specific enhancers de novo. We sample a broad set of candidate enhancer regions using data on CREB-binding protein co-factor binding or ATAC-seq chromatin accessibility, and then identify those regions with significant differences in histone acetylation between tissues. This method identifies hundreds of novel DV enhancers and outperforms ChIP-seq data of relevant transcription factors when benchmarked with mRNA expression data and transgenic reporter assays. These DV enhancers allow the de novo discovery of the relevant transcription factor motifs involved in DV patterning and contain additional motifs that are evolutionarily conserved and for which the corresponding transcription factors are expressed in a DV-biased fashion. Finally, we identify novel target genes of the regulatory network, implicating morphogenesis genes as early targets of DV patterning.

**Conclusions:**

Taken together, our approach has expanded our knowledge of the DV patterning network even further and is a general method to identify enhancers in any developmental system, including mammalian development.

**Electronic supplementary material:**

The online version of this article (doi:10.1186/s13059-016-1057-2) contains supplementary material, which is available to authorized users.

## Background

Identifying and deciphering the function of *cis*-regulatory enhancers in the genome is a major challenge but has become an attainable goal over the past decade thanks to genomics approaches. An intrinsic difficulty is that enhancer function during development is highly stage- and tissue-specific, which makes it challenging to obtain sufficient material from developing embryos for analysis. Model organisms such as *Drosophila,* for which development is well-studied and large amounts of cells can be obtained, are therefore an excellent system to test our ability to identify enhancers involved in embryonic development. Dorso-ventral (DV) pattern formation in the early *Drosophila* embryo is a good example. DV patterning is one of the earliest patterning processes in the metazoan embryo [1], relevant to understanding early pattern formation and morphogenetic movements, including gastrulation. As a result of systematic genetic screens in the 1980s, molecular analysis in the 1990s, and genomic approaches in the 2000s, it is one of the best-understood developmental networks to date. Importantly, the DV patterning system is experimentally accessible due to well-characterized mutants.

In *Drosophila,* DV patterning sets up the initial germ layers: mesoderm on the most ventral side, neurectoderm in the middle, and dorsal ectoderm on the dorsal side, which also gives rise to the extraembryonic amnioserosa most dorsally. Patterning these tissues requires the graded activity of two conserved signal transduction pathways, the Toll (Tl) and bone morphogenetic protein (BMP) signaling pathways.

Maternally deposited signals activate the Tl signaling pathway on the ventral side and lead to high nuclear concentrations of the NF-kB transcription factor Dorsal (Dl) [2]. High levels of nuclear Dl then induce the expression of two additional transcription factors, Twist (Twi) and Snail (Sna) [3–5], which together specify mesodermal fate on the ventral side. Dl also represses *decapentaplegic* (*dpp*), which encodes the main *Drosophila* BMP homolog [6], and *zerknüllt* (*zen*), which encodes a homeodomain transcription factor [7, 8]. Due to ventral repression, *dpp* and *zen* are expressed on the dorsal side and specify the amnioserosa and the dorsal ectoderm [9–11]. Dpp activity on the dorsal side activates the transcription factor Mothers against dpp (Mad) [12].

Over the years, many enhancers have been characterized that are differentially activated along the DV axis, and their identification reflects the progress in technology. Initially, enhancers were identified by testing DNA near the promoters of known DV target genes for regulatory activity in *lacZ* reporter assays [3, 4, 6, 8] or by testing DNA regions located near a *lacZ* enhancer trap reporter with DV expression [13]. Additional DV enhancers were identified by bioinformatics searches for regions with multiple transcription factor binding motif occurrences [14, 15].

The advent of genomics approaches greatly accelerated the identification of putative DV enhancers, in part due to the availability of mutant lines where all embryos in the progeny consist of either mesodermal, neurectodermal, or dorsal ectodermal precursor cells (*Tl*^*10b*^*, Tl*^*rm9/rm10*^, and *gd*^*7*^), yielding sufficient amounts of cells for genomics assays. Early expression-based methods identified novel DV target genes and enhancers by analyzing differences in mRNA levels between these DV mutants [16, 17]. Subsequent ChIP-chip and later ChIP-seq experiments enabled the systematic identification of regions that are bound by DV transcription factors in vivo, and many of these regions were confirmed as DV enhancers by *lacZ* reporter assays [18–20].

Despite the general success of transcription factor ChIP-seq experiments in identifying enhancers, there are some caveats to the approach. First, transcription factor occupancy can only be obtained if the relevant transcription factors are known and specific antibodies are available. In less well-studied systems or model organisms, this can be a significant experimental obstacle. Second, transcription factors occupy a large number of putative enhancers and have an uncertain number of false positives. For example, the Twi and Sna ChIP signal can be detected at thousands of putative regulatory regions [18–21], but genetics and gene expression data predict a much smaller number of target genes [16]. While bona fide enhancers tend to have a high ChIP signal [19, 22], not all Twi regions that were selected based on high ChIP signal were confirmed in transgenic reporter assays [19, 20].

An emerging realization is that the binding of transcription factors, as well as those of co-activators, occurs to some degree promiscuously at regions of open chromatin and may not necessarily indicate regulatory activity [22–25]. This conclusion is consistent with imaging studies, suggesting frequent non-specific collisions of transcription factors with DNA [26, 27] and thus questions the reliability of transcription factor occupancy for enhancer identification. However, in the absence of a gold standard, it is difficult to systematically evaluate the strengths and weaknesses of various methods.

As an alternative to transcription factor occupancy, acetylation of lysine 27 on histone H3 (H3K27ac) has been shown to be a reliable marker for active enhancers in mammalian systems [28–30] and in *Drosophila* [31]. However, methods by which H3K27ac could be systematically used to identify enhancers in a developmental context have not yet been explored in detail. Such analysis is not straightforward, because H3K27ac is found in broad regions flanking active enhancers and is not found at active enhancers themselves, which are depleted of nucleosomes [28, 31]. How to best identify putative regions that might represent active enhancers and how to use H3K27ac ChIP-seq data as a measurement for tissue-specific activity in the embryo is therefore not entirely clear.

Here we used the well-studied DV patterning system to determine how to best use H3K27ac data to identify tissue-specific enhancers. We find that a highly effective method is to first use an independent dataset to identify candidate enhancer regions and then to specifically query these regions for differential H3K27ac across tissues. We show that candidate enhancer regions can be identified with ChIP-seq data of the co-activator CREB-binding protein (CBP or Nejire in *Drosophila*) or the assay for transposase-accessible chromatin using sequencing (ATAC-seq) [32].

Using this approach, we identified several hundred putative enhancers for mesoderm and dorsal ectoderm that we validated with expression data, transgenic reporter assays, and transcription factor motif analyses. Notably, the regions identified by differential H3K27ac analysis were more accurate in their expected motif content and tissue specificity compared to regions identified by transcription factor occupancy (ChIP-chip or ChIP-seq).

With these newly identified DV enhancers, we were able to identify de novo the motifs of most known DV transcription factors and show that these motifs are highly conserved across species. Furthermore, we identify novel DV target genes, including transcription factors and signaling components, as well as genes involved in morphogenesis that may function in early tissue movements, including gastrulation. Since our approach is a general method to identify enhancers that are differentially active between tissues, it should be applicable to any developmental system, including vertebrate tissues.

## Results

### Known DV enhancers are marked by relative differences in H3K27ac across tissues

To identify novel DV enhancers using H3K27ac, we focused on the most ventral and the most dorsal tissue by using *Tl*^*10b*^ embryos and *gd*^*7*^ embryos. *Tl*^*10b*^ embryos have uniformly high Dl activity throughout the embryo and thus represent the presumptive mesoderm on the ventral side [33]. *gd*^*7*^ embryos completely lack Dl activation and represent the presumptive dorsal ectoderm on the dorsal side [34]. We then performed replicate ChIP-seq experiments for H3K27ac and mRNA-seq in each DV mutant during the time in which DV patterning takes place (2–4 h after egg deposition (AED), corresponding to embryonic stages 5–9). Replicate experiments were highly reproducible (Additional file 1: Supplementary material).

To explore how H3K27ac could be used to identify novel DV enhancers, we first analyzed the pattern of H3K27ac at known DV enhancers. We defined known DV enhancers as those for which DV-specific reporter gene expression has been reported in the literature (see Additional file 2: Table S1 and Additional file 1: Supplementary material). Since the known enhancers vary in size dependent on how they were discovered, we measured H3K27ac ChIP-seq enrichment for each enhancer within a fixed 1-kb window centered on the enhancer’s midpoint. This window is large enough to extend beyond the nucleosome-depleted enhancer region, thus sampling the flanking H3K27ac levels, but is small enough to avoid sampling potential H3K27ac enrichment from other enhancers nearby. Thus, the 1-kb window is sufficient to detect changes in H3K27ac while maximizing resolution, i.e., to distinguish between the activity of two neighboring enhancers.

**Fig. 1:**
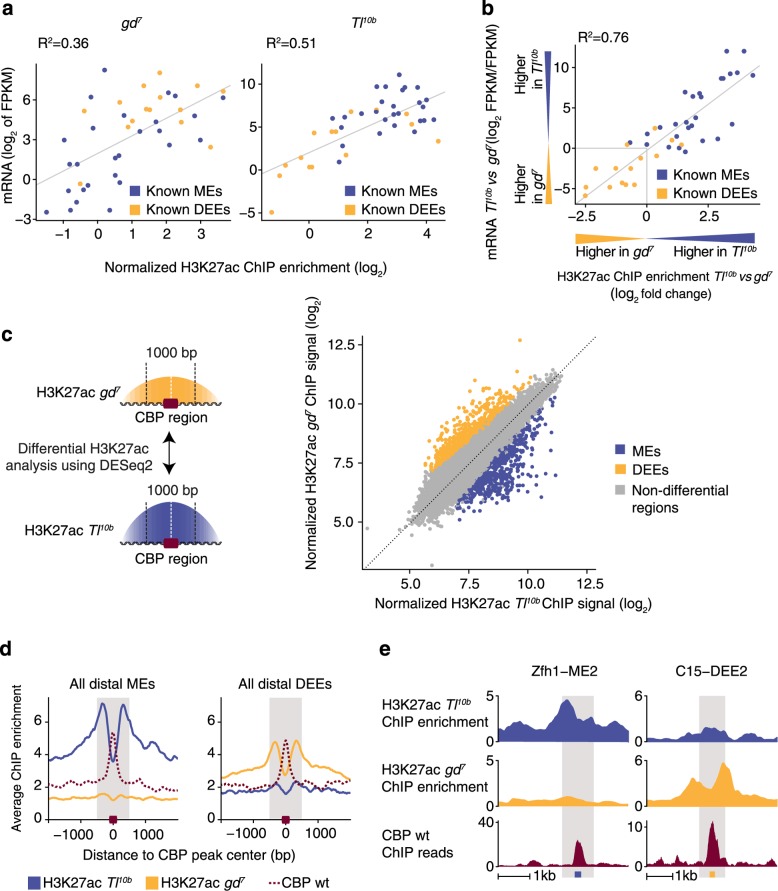
Differential H3K27ac analysis across tissues is an effective method to identify tissue-specific enhancers. **a** Scatterplots of H3K27ac ChIP-seq enrichment at each known DV enhancer (1 kb centered on midpoint) against the transcript levels of the known target gene show a good but modest correlation in dorsal ectoderm precursor embryos *gd* ^*7*^ (*left*) and in mesoderm precursor embryos *Tl* ^*10b*^ (*right*). ChIP-seq enrichment values represent fold change over the corresponding input control after normalizing for differences in read count and fragment size. *MEs* mesoderm enhancers, *DEEs* dorsal ectoderm enhancers. **b** The correlation between H3K27ac and transcript levels becomes stronger when the fold changes in H3K27ac levels between the two mutant embryos at each enhancer are plotted against the fold changes in transcript levels of the corresponding target genes. See Additional file 3: Figure S1 for gene names. **c** De novo identification of DV enhancers based on CBP candidate regions and differential H3K27ac analysis by the package DESeq2. Numerous candidate enhancers were located with CBP ChIP-seq data in wild-type embryos. DESeq2 was then used to identify significant differences in H3K27ac between *gd* ^*7*^ and *Tl* ^*10b*^ embryos within 1-kb windows centered on the CBP peaks. The average DESeq2-normalized ChIP signal for all replicates is shown as a scatterplot on the *right*. CBP regions significantly different for H3K27ac are shown in *blue* (MEs) and *yellow* (DEEs), while non-differential regions are shown in *gray*. **d** Average enrichment of H3K27ac in *gd* ^*7*^ and *Tl* ^*10b*^ embryos, as well as CBP in wild-type embryos, is shown for MEs and DEEs that were located distally, at least 1 kb from any transcription start site (*TSS*). The *gray bar* represents the 1-kb window used to calculate H3K27ac enrichments, and the *red box* represents the 201-bp enhancer region. **e** Examples of newly identified distal enhancers zfh1-ME2 and C15-DEE2. H3K27ac is shown as normalized ChIP enrichments over input, while CBP is shown as normalized ChIP reads (reads per million). The *gray bar* represents the 1-kb H3K27ac window, and the *blue* and *yellow boxes* represent the 201-bp ME or DEE enhancer region, respectively

We first tested whether the H3K27ac enrichment levels at each enhancer could be used as an absolute marker for enhancer activity in each tissue. By using the transcript levels of the corresponding target genes as a proxy for each enhancer’s activity, we found a reasonably high correlation between H3K27ac enrichment and enhancer activity in both tissues (*R*^2^ = 0.36 and *R*^2^ = 0.51, Fig. 1a, see Additional file 3: Figure S1 for gene names). This general trend is consistent with previous reports [28–30].

Although H3K27ac was overall a good marker for active enhancers, H3K27ac enrichment values are found in a continuum, and high H3K27ac enrichment in one tissue was not a good marker for its tissue-specific activity (Fig. 1a). For example, active enhancers tend to have higher H3K27ac enrichments; however, within the same tissue, H3K27 enrichments are not always higher at active compared to inactive enhancers (the yellow and blue dots are not separated in Fig. 1a).

We therefore tested whether the relative difference between H3K27ac at each enhancer across tissues would be a better measurement for the tissue specificity. Indeed, when we plotted the relative difference in transcript levels against the relative difference in H3K27ac levels for all enhancers, the tissue specificity of each enhancer became apparent. The majority of enhancers showed both higher H3K27ac and higher transcript levels in the expected tissue (the blue and yellow dots are much more separated from each other in Fig. 1b). Strikingly, this also improved the overall correlation (*R*^2^ = 0.76, Fig. 1b). This suggests that analyzing the absolute levels of histone modifications is useful, but that in a typical biological setting, a more effective way of identifying tissue-specific enhancers is to focus on relative differences in H3K27ac between tissues.

We therefore used relative H3K27ac differences between tissues to identify putative DV enhancers. When applied across the genome, differential H3K27ac analysis also has the advantage of identifying only tissue-specific enhancers, while excluding enhancers that are equally active in both tissues.

### Identification of candidate enhancer regions through CBP ChIP-seq or ATAC-seq

So far we used the location of known DV enhancers to measure relative differences in H3K27ac levels. To identify DV enhancers throughout the genome de novo, we could systematically analyze any differences in H3K27ac between tissues. However, since the peak levels of H3K27ac are found next to enhancers, this approach would not identify the exact location of putative DV enhancers. We therefore decided to systematically map the location of putative enhancer regions first and to specifically test these regions for significant differences in H3K27ac levels within a 1-kb window (Fig. 1c).

To identify candidate regions, we did not use transcription factor ChIP-seq data, since they could introduce a bias towards a specific set of target genes and potentially a specific tissue, which we wished to avoid. Furthermore, we wanted to develop a method that would be generally applicable to any developmental system, without knowledge of the transcription factors involved.

A commonly used marker for enhancers is the co-factor CBP [28, 35, 36]. Although its binding is not restricted to enhancers [37], CBP is the main histone acetyl transferase that catalyzes H3K27ac in *Drosophila* [38]. Therefore, regions with CBP binding should serve as excellent candidates for querying differential H3K27ac across the genome. To test this approach, we performed CBP ChIP-seq experiments on *Drosophila* embryos (2–4 h AED) and identified 15,477 candidate enhancer regions.

Another approach is to use regions of open chromatin as candidate enhancer regions. Chromatin accessibility is thought to be a general feature of enhancers and can be measured in any developmental system without the need for specific antibodies. A recently developed assay, ATAC-seq, can be used for this purpose with a relatively small number of cells and thus should be particularly attractive for developmental systems. We therefore performed ATAC-seq on *Drosophila* embryos (2–4 h AED) and identified 29,510 accessible regions.

We next tested the suitability of the CBP and ATAC regions as candidate enhancers and found that CBP regions overall performed slightly better. First, CBP regions covered more known DV enhancers (57 out of 59) than did ATAC regions (51 out of 59). Second, when we compared these regions to Vienna Tiles (VTs) that have been documented to drive transgenic reporter gene expression [39], we found that tiles overlapping the top 10,000 CBP regions were more frequently annotated with early embryo expression patterns than tiles overlapping the top 10,000 ATAC regions (52 % among CBP regions versus 33 % among ATAC regions), suggesting that a greater portion of CBP regions than ATAC regions are functional enhancers. Finally, we found less evidence for sequence bias among the CBP regions. Among the top 500 CBP regions, the motif of Zelda (Zld) was enriched (Additional file 3: Figure S2). This is expected, since it is the most common motif among all early enhancers [40]. Although CBP has been reported to interact with a number of transcription factors (see, e.g., [41, 42], no motifs involved in embryonic patterning were enriched among the top 500 CBP regions (Additional file 3: Figure S2), arguing against a bias towards certain signaling pathways. ATAC regions did not show any bias towards patterning transcription factors either, but a number of repetitive motifs showed significant enrichment, which could indicate a certain sequence bias in this group (Additional file 3: Figure S2).

Taken together, our analysis suggests that CBP regions are more likely to be enhancers and are less sequence-biased compared to ATAC-seq regions. We therefore present the results from using CBP regions as the starting point for our analysis. However, we have also performed the same analysis for ATAC-seq regions and found similar results (shown in Additional file 3: Figures S2–S5), suggesting that ATAC-seq regions are a useful alternative to CBP regions.

### Large-scale differential H3K27ac analysis on candidate regions reveals hundreds of putative DV enhancers

To systematically query our CBP candidate enhancer regions for significantly different H3K27ac levels between tissues, we applied the Bioconductor package DESeq2 [43]. Strikingly, hundreds of regions are significantly different between the two tissues (*q* value <0.01 after Benjamini-Hochberg correction). We obtained 594 regions with higher H3K27ac in *Tl*^*10b*^ versus *gd*^*7*^, thus putative mesoderm enhancers (MEs) (blue dots in Fig. 1c), and 572 regions with higher H3K27ac in *gd*^*7*^ versus *Tl*^*10b*^, thus putative dorsal ectoderm enhancers (DEEs) (yellow dots in Fig. 1c). Among these regions were 34 known DV enhancers, while 25 known DV enhancers were not identified as significantly different between tissues. Closer inspection revealed that the majority of known DV enhancers showed some evidence for differential H3K27ac (see also Fig. 1b), suggesting that the missed DV enhancers are not regulated in a different manner but are largely due to noise in the data and therefore did not pass our statistically stringent criteria.

We then concentrated on putative distant enhancer regions, which are at least 1 kb away from a transcription start site (TSS), since such distal enhancers have historically been more challenging to identify. Focusing on distal enhancers also avoids confounding effects from differences in transcription as well as biases in sequence motifs that are preferentially found in promoter regions. After excluding TSS regions, we obtained 416 putative MEs and 380 putative DEEs, summarized in Additional file 4: Table S2. When we assigned these putative enhancers to the nearest active gene based on mRNA-seq data, only 23 % were assigned to a unique gene, while most assigned genes had multiple putative enhancer regions (Additional file 3: Figure S6), consistent with previous findings [19, 44]. We named each putative enhancer based on the assigned gene and enhancer type and numbered them based on the location along the chromosome (e.g., C15-DEE1, C15-DEE2, and so on). For completeness, we also assembled and analyzed the list of putative enhancers that overlapped a TSS (Additional file 5: Table S3), but we present here our analysis on the distal enhancers.

As expected, the distal MEs and DEEs showed the highest levels of H3K27ac in the flanking regions, on average ~330 bp up- and downstream of the CBP peak center (Fig. 1d). Analysis of individual regions confirmed that H3K27ac peaks were typically enriched surrounding the putative enhancers we identified (e.g., zfh1-ME2 and C15-DEE2 in Fig. 1e).

### Putative DV enhancers identified by differential acetylation analysis show higher tissue specificity than those identified by transcription factor occupancy

**Fig. 2:**
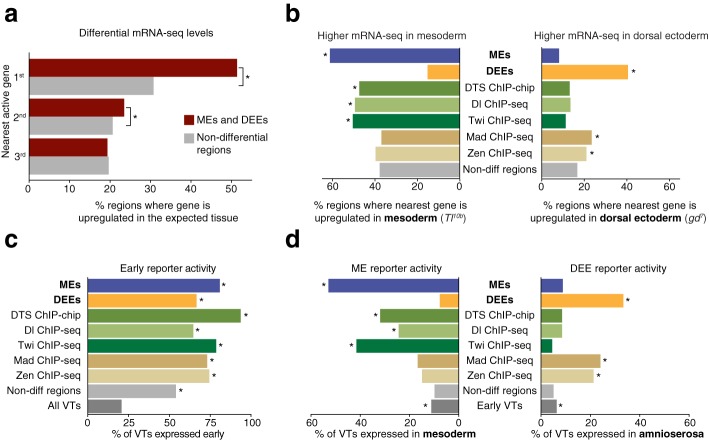
Genes near putative enhancers are differentially regulated across tissues. **a** The closest active genes near MEs and DEEs are significantly differentially expressed in the expected tissue (*Tl* ^*10b*^ or *gd* ^*7*^) as compared to non-differential control regions (*p* < 10^−30^, one-sided chi-squared test). The second closest genes also show a small but significant enrichment (*p* < 0.034, one-sided chi-squared test), while the third nearest active genes are no longer enriched over the control. **b** Fraction of genes with higher mesodermal expression (*left*) is largest among MEs, and that with higher dorsal ectoderm expression (*right*) is largest among DEEs. As a comparison, the top 400 regions identified by transcription factor ChIP-seq data are shown: Dl and Twi ChIP-seq experiments from *Tl* ^*10b*^ embryos, and Mad and Zen ChIP-seq experiments from *gd* ^*7*^ embryos. The *star* marks significance over non-differential regions (*p* < 0.01, one-sided chi-squared test). **c** Transgenic reporter activity of Vienna Tiles (*VTs*) that overlap MEs, DEEs, and transcription factor ChIP regions are preferentially expressed during early embryonic stages (with annotated expression in any tissue at stages 4–10). All groups show expression far above the average of all VTs (marked as *star*, *p* < 0.01, Fisher’s exact test). **d** VT reporter expression is more tissue-specific for MEs and DEEs compared to transcription factor ChIP regions. ME reporter activity (*left*) was defined as annotations by Kvon et al. [39] containing “mesoderm,” and DEE reporter activity (*right*) as annotations containing “amnioserosa.” The *star* marks significance over non-differential regions (*p* < 0.01, Fisher’s exact test). Number of regions: all VTs (7705), early expressed VTs (1595), VTs overlapping putative DV enhancers (148), MEs (68), DEEs (80)

To validate the identified MEs and DEEs, we first analyzed whether the mRNA-seq levels of the nearby genes were significantly higher in the expected tissue (e.g., whether the mRNA transcripts near MEs are significantly higher in *Tl*^*10b*^ over *gd*^*7*^). As a control group, we used the CBP regions without differential H3K27ac (”non-differential regions”, *n* = 6352). We found that 51 % of the closest active genes are differentially expressed in the predicted tissue, significantly higher than in the control group of CBP regions, which have 31 % (*p* < 10^−30^, Fig. 2a). The second nearest active genes were also slightly more differentially expressed (*p* < 0.034) than the control, but the third nearest genes were no longer enriched, consistent with enhancers functioning predominately locally in *Drosophila* [39]. Overall, we found that a large fraction of putative DV enhancers is associated with gene activation in the expected tissue.

To test whether our approach performs better than previous methods, we performed the same analysis using putative DV enhancers identified previously with ChIP-chip experiments of Dl, Twi, and Sna in *Tl*^*10b*^ embryos (high confidence Dl Twi Sna (DTS) non-TSS regions, *n* = 213, Zeitlinger et al. [19], obtained from http://younglab.wi.mit.edu/dorsal/Dorsal_network_targets.txt). We also performed ChIP-seq experiments of Dl and Twi in *Tl*^*10b*^ embryos as well as ChIP-seq experiments of Mad and Zen in *gd*^*7*^ embryos. Although thousands of regions were significantly bound in each ChIP-seq sample (Additional file 6: Table S4), we selected the top 400 regions based on highest confidence. In this manner, the number of regions in the transcription factor groups are similar to those identified by differential H3K27ac and are likely of high quality, since known DV enhancers tend to have higher occupancy of Dl, Twi, and Sna than other regions [19].

We found that the fraction of regions for which the nearest active gene showed differential expression was higher for the MEs and DEEs identified by differential H3K27ac than for the regions identified by transcription factor occupancy in the corresponding tissue (Fig. 2b). The result was particularly striking for DEEs, since regions identified by Mad or Zen binding were only half as likely as DEEs to have a nearby gene with higher expression in the dorsal ectoderm (Fig. 2b).

To more directly evaluate the ability of our MEs and DEEs to drive reporter activity in the correct tissue, we took advantage of the large-scale analysis of Vienna Tiles (VTs) [39]. VTs are 2-kb non-coding genomic fragments that have been analyzed for their ability to drive transcription of a transgenic reporter during *Drosophila* embryogenesis. There are 148 of these VTs that overlap with our identified MEs and DEEs.

We first calculated the fraction of these VTs that drive reporter activity in any tissue during early embryogenesis. We found that among VTs that overlap MEs, DEEs, or the 400 top regions for each transcription factor, at least 65 % had early reporter gene activity in each group (Fig. 2c), significantly above the 20 % average of all VTs (Fig. 2c). This suggests that differential acetylation and transcription factor occupancy are both successful in identifying bona fide enhancers.

There was, however, a noticeable difference in the degree of tissue specificity between the groups (Fig. 2d). Among the 68 VTs overlapping our identified MEs, 53 % have mesodermal tissue expression annotations, as determined by Kvon et al. [39]. In comparison, only 10 % of non-differential control regions had such annotations (Fig. 2d, *p* < 10^−24^). Among ChIP regions, those identified with mesodermal transcription factors (DTS, Dl, and Twi) also drove reporter activity in the mesoderm more often than the non-differential control regions, but the percentages were not as high as for the newly identified MEEs (32 %, 24 %, and 42 %, respectively, Fig. 2d).

Similarly, 33 % of VTs that overlap our identified DEEs have annotated expression in the amnioserosa (a dorsal tissue that can easily be queried). In comparison, 5 % of VTs of non-differential control regions show this annotation (*p* < 10^−19^). The ChIP-based regions identified through transcription factors active in the dorsal ectoderm (Mad and Zen) also performed significantly above the control (24 % and 21 %, respectively) but not as highly as the DEEs (33 %) (Fig. 2d).

This suggests that transcription factor ChIP regions are highly enriched for enhancers, but that even highly bound regions may not be active in the tissue analyzed. Overall, differential H3K27ac analysis is very reliable in identifying enhancers with differential activity in the examined tissues.

### Putative DV enhancers are enriched for expected transcription factor motifs

Our putative MEs and DEEs were not based on prior knowledge of the DV transcriptional network, and thus we used this opportunity to ask whether we could rediscover the known *cis*-regulatory motifs among these sequences. To perform a comprehensive motif analysis, we collected all known *Drosophila* transcription factor motifs from FlyFactorSurvey [45] and JASPAR [46] and used FIMO [47] to score their presence in all ME and DEE sequences (201 bp centered at the CBP peak).

**Fig. 3:**
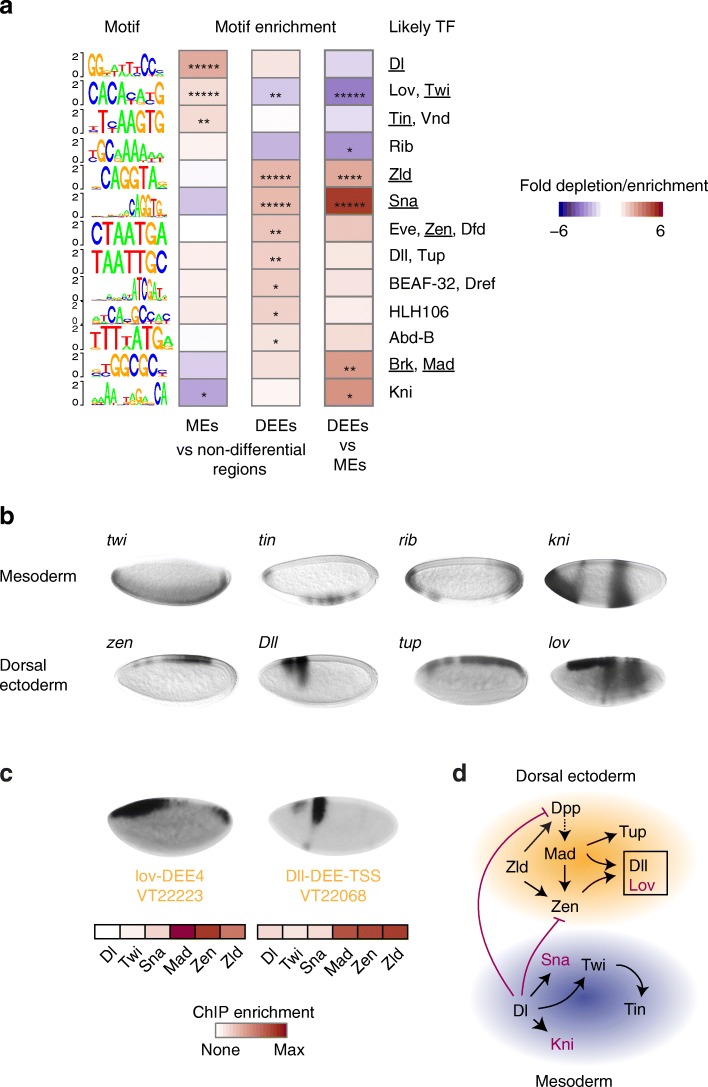
The identified putative DV enhancer regions are enriched for known DV transcription factor motifs. **a** Known *Drosophila* motifs that are significantly enriched (*red*) or depleted (*blue*) at MEs or DEEs over non-differential control regions, or DEEs over MEs, are shown as sequence logos in bits on the *left*. The transcription factor that is known to bind the motif is shown on the *right*. If there are several transcription factors (*TFs*), all matches are shown, and the factor that is known to function during DV patterning is *underlined*. Significance was determined by a one-sided proportion test (* *p* < 0.05, ** *p* < 10^−2^, *** *p* < 10^−3^, **** *p* < 10^−4^, ***** *p* < 10^−5^ after Benjamini-Hochberg correction). **b** Many identified transcription factors are expressed in a DV-specific pattern themselves, e.g., localized to the mesoderm (*top*) or the dorsal ectoderm (*bottom*) in in situ hybridization images from stage 4, obtained from the Berkeley *Drosophila* Genome Project (BDGP) in situ hybridization database [73–75]. Among them are *lov* and *Dll*, which are expressed in the dorsal ectoderm. **c** Expression of *lov* and *Dll* in the dorsal ectoderm could be mediated by two enhancers identified among DEEs (lov-DEE4 and Dll-DEE-TSS). Overlapping VTs drive reporter activity similar to the endogenous genes. Both enhancers are occupied by Mad, Zen, and Zld based on ChIP-seq data, indicating direct regulation by the DV network. Dl, Twi ChIP-seq are from *Tl* ^*10b*^ embryos, Sna ChIP-seq from wild-type embryos, and Mad, Zen, Zld ChIP-seq from *gd* ^*7*^ embryos. **d** DV regulatory network based on the rediscovered transcription factors and lov and Dll as added components (*boxed*). The shown regulatory interactions are based on literature knowledge, confirmed by our own ChIP-seq data. Shown in *red* are transcription factors that likely function as repressors, since the tissue in which they are expressed is distinct from the tissue of their target genes

We then scored the relative motif enrichments among MEs or DEEs over non-differential H3K27ac control regions, or DEEs over MEs using a Fisher exact test (*p* < 0.05 after Benjamini-Hochberg correction, Fig. 3a). To avoid false positives, we only scored motifs for which the transcription factor was expressed in either the mesoderm (*Tl*^*10b*^) or dorsal ectoderm tissue (*gd*^*7*^). We then collapsed motifs whose occurrences substantially overlapped (see Methods). In these cases, we show the most significant motif with the corresponding transcription factor, as well as other transcription factors if they have known roles in DV patterning. In total, we found 13 independent motifs that were significantly enriched in our putative DV enhancers (Fig. 3a).

Our unbiased motif discovery captured motifs for all well-established DV transcription factors. Among MEs, motifs for Dl and Twi were enriched, as expected, as well as the motif for Tinman (Tin), a transcription factor that is known for its role in muscle development [18, 48, 49].

Among DEEs, we found the motifs for Zen, Zld, and Brinker (Brk), as expected. The Brk motif is bound by Mad, the downstream nuclear target of the Dpp signaling pathway, in competition with Brk [50–53]. The Zen motif was similar to other homeodomain motifs, including that of Even-skipped (Eve), which scored even higher than that of Zen.

Among DEEs, we also identified the Sna motif as highly enriched. Since Sna is expressed in the mesoderm, this suggests that Sna represses dorsal ectodermal genes. Indeed, while Sna is better known for repressing neurectodermal genes [5], Sna has also been shown to repress the dorsal ectodermal gene *pannier (pnr)* [54]. Furthermore, highly conserved Sna motifs have previously been found in putative dorsal ectodermal enhancers [19].

To test whether our identified motifs are indeed bound by the expected DV transcription factors in vivo, we analyzed the motifs’ enrichments in the respective ChIP-seq data from Dl, Twi, Sna, Zen, Mad, and Zld. We found that MEs and DEEs with the motifs showed enriched ChIP occupancy of the corresponding transcription factor compared to MEs and DEEs without the motif (Additional file 3: Figure S7). It remains to be shown whether other transcription factors known to bind similar motifs in vitro also bind to these motifs in vivo, perhaps in competition with the known DV transcription factors.

Among the remaining motifs, a large fraction of associated transcription factors are themselves expressed in a DV-biased fashion (*ribbon (rib), knirps (kni)* on the ventral side*, Distal-less* (*Dll), tail-up (tup), jim lovell (lov,* also known as CG16778) on the dorsal side, Fig. 3b), and some of them have known roles in early development. *rib* is expressed mostly at the poles and in some ventral cells and encodes a Bric-a-brac, Tramtrack, Broad (BTB) domain transcription factor with known roles in morphogenesis [55]. *kni* is expressed at the most anterior ventral cells and plays a role during head development [56], in addition to its well-known role as gap gene. The enhancer that mediates the anterior ventral expression pattern of *kni* [19, 57] was among our putative MEs (kni-ME5), confirming that *kni* is part of the DV network.

On the dorsal side, the *zen* expression pattern (stage 5) is similar to that of *tup*, a known target of Dpp [19, 58] that encodes another homeodomain transcription factor essential for amnioserosa development [59]. A third homeodomain transcription factor, Dll, is expressed in a subset of two narrow stripes on the dorsal side, in areas where *zen* and *tup* appear to be low (Fig. 3b). A motif most similar to the Dll motif has previously been shown to be essential for the expression of a dorsal ectodermal enhancer (TTAATTGC in the *pnr* enhancer [54]), consistent with Dll being part of the DV network. Finally, *lov* is specifically expressed on the dorsal side of the embryo (Fig. 3b) [60]. It encodes a BTB domain transcription factor that recognizes a motif very similar to that of Twi in vitro [45]. It is therefore conceivable that it represses Twi targets on the dorsal side.

While most of the transcription factors that we identified in our motif analysis are already known targets of the DV network, Rib, Dll and Lov are not. If these genes are in fact involved in DV patterning, we would expect them to have DV-regulated enhancers themselves. Indeed, among our MEs and DEEs, we found putative enhancers for *lov* and *Dll* that had the characteristics of typical DV enhancers (Fig. 3c). In each case, the DEE overlapped with a VT that drove expression in the appropriate DV pattern and showed high occupancy of Mad and Zen. We therefore added Lov and Dll to the known DV network (Fig. 3d).

As a control, we also performed the same motif analysis on the putative enhancer regions identified by transcription factor occupancy. While we identified a large number of enriched motifs, the motifs were surprisingly different between all transcription factors and were hard to interpret, especially with regard to tissue specificity (Additional file 3: Figure S2). This further corroborates our conclusion that transcription factor occupancy is not ideal for identifying tissue-specific enhancers.

### Phylogenetic conservation of motifs among putative DV enhancers

**Fig. 4:**
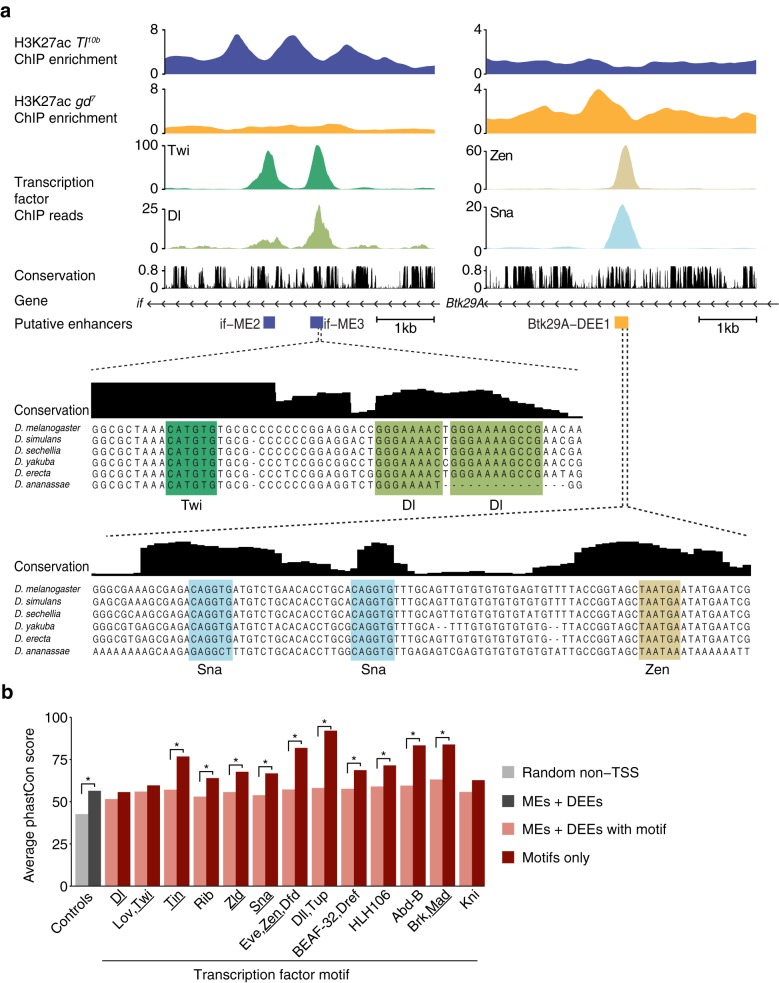
Transcription factor binding motifs are conserved in novel DV enhancers. **a** Examples of conserved motif instances within a mesoderm enhancer (if-ME3, *left*), close to the gene *inflated (if)*, and a dorsal ectoderm enhancer (Btk29A-DEE1, *right*), close to the *Btk29A* gene. H3K27ac is shown as normalized ChIP enrichment over input in *Tl* ^*10b*^ and *gd* ^*7*^ embryos. ChIP-seq occupancy is shown for Twi and Dl (*left*) and Zen and Sna (*right*) as ChIP-seq reads normalized to reads per million. A close-up of the if-ME3 sequence shows a canonical Twi binding motif (*E-box*) and two Dl binding sites that are conserved across several *Drosophila* species. A close-up of the Btk29A-DEE1 sequence shows two canonical Sna binding motifs and a canonical Zen binding motif that reside in islands of conservation. Conservation data are phastCons data obtained from the UCSC genome browser [72]. **b** Conservation of all identified DV motifs among all MEs and DEES. The average phastCons score for all putative DV enhancers (”MEs + DEEs” in *dark gray*) is significantly higher compared to control regions (”Random non-TSS” in *light gray*). The average phastCons score of each DV motif (”Motifs only” in *red*) is in many cases higher than that of the surrounding regions (”MEs + DEEs with motif” in *light red*). This confirms that motifs like that of Zen and Sna are indeed preferentially found in islands of conservation. Significance was determined by Wilcoxon rank-sum test and marked with a *star* (*p* < 0.05)

Phylogenetic sequence conservation (“phylogenetic footprinting”) of regulatory regions, and specifically of the transcription factor binding motifs within them, has long been used to identify functional enhancers and motifs [61, 62]. When we analyzed the newly identified enhancers, we noticed that the identified transcription factor motifs were often found within sequence blocks of high conservation across the *Drosophila* phylogeny, interspersed by more diverged sequences within the same enhancer region. For example, a canonical Twi binding motif in the newly identified if-ME3 enhancer is in a highly conserved sequence block, while a partially conserved sequence block right next to it contains two Dl binding motifs (Fig. 4a left). Likewise, a putative DEE, Btk29A-DEE1, has two conserved, presumably repressive, Sna binding motifs next to a conserved canonical Zen binding motif, each in a conserved sequence block (Fig. 4a right).

We therefore tested more systematically whether the identified motifs tended to be among blocks of conservation (Fig. 4b). As a baseline control, we first calculated the average phastCons score of all MEs and DEEs and found it to be significantly above that of random non-TSS regions (Fig. 4b, 56 % ”MEs + DEEs” versus 43 % ”Random non-TSS,” *p* < 10^−43^), showing that ME and DEE 201-bp sequences are evolutionarily conserved above average. We then calculated the average phastCons score for each identified DV motif among all MEs or DEEs (Fig. 4b ”Motifs only”) and compared it to the average phastCons score for all MEs and DEEs where the motifs were found (Fig. 4b ”MEs + DEEs with motif”). This allowed a direct comparison between the conservation of the motif and that of its surrounding regions.

This analysis shows that the motifs found in putative DV enhancers (”Motifs only”) for Tin, Rib, Zld, Sna, Eve, Dll, BEAF-32, HLH106, Abd-B, and Brk are significantly more conserved than their surrounding regions ”MEs + DEEs with motif” (Fig. 4b), which are already conserved above average. The highest conservation was found for the homeodomain motifs (Eve, Dll, Abd-B) and the Brk motif (Fig. 4b), which suggests a prominent and ancient role of homeodomain transcription factors in the development of dorsal ectoderm. Taken together, these results further validate our method to identify enhancers based on differential acetylation.

### Novel DV target genes involved in morphogenesis

Our list of newly identified MEs and DEEs is highly enriched for bona fide DV target enhancers based on independent assays such as mRNA expression and transgenic reporters. Yet, these putative DV enhancers may contain false positives, and thus we focused on high-confidence DV enhancers and their potential target genes to gain a better understanding of all the genes that are targeted by the DV network.

**Fig. 5:**
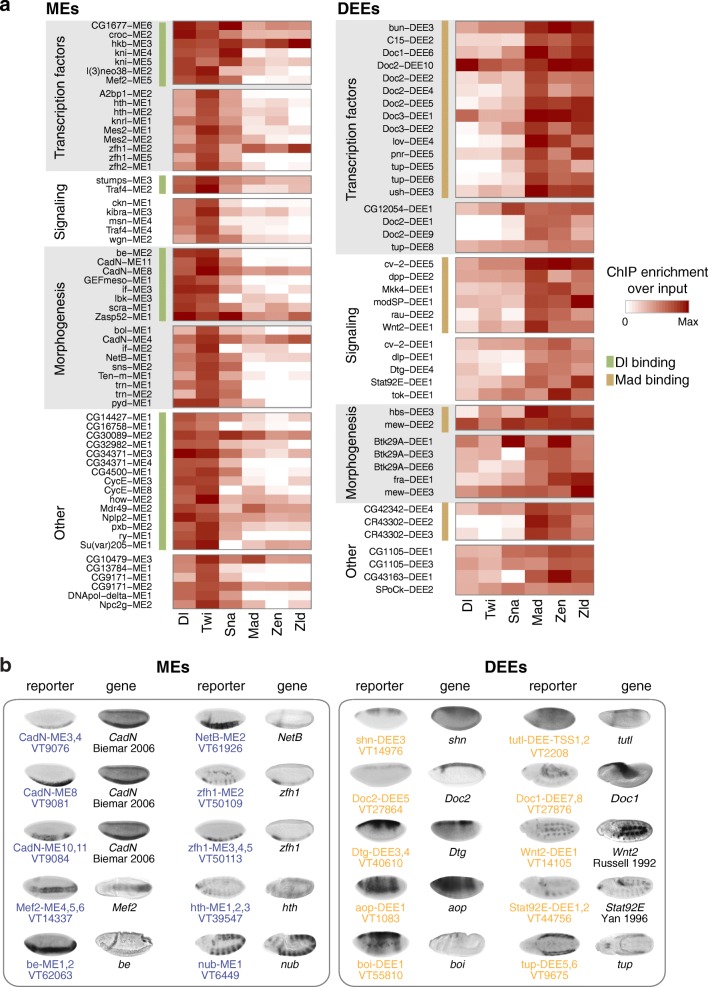
High-confidence DV enhancers identify novel DV target genes. **a** All MEs and DEEs that were confirmed by high occupancy of DV transcription factors and differential target gene expression by mRNA-seq. The occupancy of DV transcription factors Dl, Twi, Sna, Zen, Mad, and Zld is shown as a heatmap of normalized ChIP enrichment over input. The enhancers were categorized based on the function of their target genes (transcription factors, signaling, morphogenesis, other) and whether they have high occupancy of Dl, Mad (*green* and *light brown bars*, respectively), which indicates that they are likely direct targets of the signaling cascade. **b** A selection of MEs and DEEs that were confirmed by in vivo reporter expression of overlapping Vienna Tiles (*VTs*), which match the expression pattern of the assigned target gene. In situ hybridization images for VTs were obtained from Kvon et al. [39]. Unless noted otherwise, the in situ hybridization images for target genes are from the BDGP database [73–75]. The *CadN* expression pattern is by Biemar et al. [17], copyright (2006) National Academy of Sciences, USA; that of *be* is from Fly-FISH [76, 77] and was color-modified to resemble the black-and-white in situ hybridization images, reused with permission; that of *Wnt2* is from Russell et al. [78] Copyright 1992 The Company of Biologists, and that of *Stat92E* is from Yan et al. [79], reprinted from Yan et al., Identification of a *Stat* gene that functions in *Drosophila* development. *Cell*, 84(3):421–430, Copyright 1996, with permission from Elsevier

First, we assembled all putative DV enhancers that have high ChIP-seq occupancy of DV transcription factors (Dl, Twi, Mad, or Zen) and have a nearby gene that is upregulated in the expected tissue based on mRNA-seq data. We then grouped the enhancers based on their pattern of DV transcription factor occupancy and the tissue in which they are active (Fig. 5a). Second, we assembled novel DV enhancers that overlapped a VT whose expression pattern matched that of the assigned nearby gene (Fig. 5b).

Among these high-confidence DV enhancers, a large fraction of the target genes encode transcription factors and signal transduction molecules, many of which were previously known or have fitting roles in DV patterning (e.g., *cv-2, Dtg, tok* in the Dpp signaling pathway). However, we also identified a number of genes that likely play a role in the morphogenesis of the developing tissues, including genes regulating the cytoskeleton or cell adhesion (Fig. 5a). Many of these novel putative morphogenesis genes have high Dl occupancy (*be, GEFmeso, if, lbk, scra, Zasp52)*, suggesting that they are directly activated by Dl on the ventral side and thus could play a role in initiating the gastrulation movements. Interestingly, *mew* is among the putative morphogenesis genes that is repressed by Dl and expressed on the dorsal side. *mew* and *if* are both alpha integrins but with different specificities in the extracellular domain [63], suggesting that their early differential DV expression may confer the developing tissues’ different cellular properties. Taken together, the identified DV enhancers provide novel target genes of the DV network, including genes that may mediate the differential cellular behavior of the analyzed tissues.

## Discussion

We showed that large-scale selection of candidate regions in combination with differential H3K27ac analysis between tissues is a simple and very effective technique for the identification of tissue-specific enhancers. Our approach did not require knowledge of relevant transcription factors and even performed better than ChIP-seq data of such factors. While regions identified by transcription factor occupancy were equally enriched for enhancers, they were more likely to drive expression in neighboring tissues than those identified through differential acetylation analysis. This was not due to technical limitations, since the ChIP-seq data are of high quality and the transcription factors are genetically required for DV patterning.

We argue that histone acetylation more accurately reflects an enhancer’s activity than the binding of transcription factors. This happens because high transcription factor occupancy is sometimes found at enhancers that are not active, presumably due to repression [18, 19]. Furthermore, histone acetylation can be measured in all tissues and thus allows the detection of relative differences between tissues, while transcription factor binding can only be measured in the tissue in which the transcription factor is expressed.

Analyzing relative differences in H3K27ac between tissues provides several benefits over analyzing each tissue separately. First, differential enhancer activity is likely more biologically relevant than the analysis of individual tissues. Traditionally, differential gene expression (e.g., visualized by mRNA in situ hybridization), rather than absolute gene expression levels, has been regarded as a hallmark of tissue patterning during development. Second, differential H3K27ac analysis specifically identifies enhancers that are different between tissues and disregards enhancers that are equally active in both tissues. In our system, this enabled us to specifically identify enhancers that are part of the Dl patterning network, including downstream transcription factors, signaling pathways, and morphogenesis genes. Third, differential H3K27ac analysis allows, in principle, the identification of enhancers that are active in only a subset of a tissue (e.g., *kni* and *Dll*). In this case, the relative difference between tissues is smaller but can still be detected if within the sensitivity of the assay.

While differential acetylation analysis has clear advantages, it also has limitations. Since histone acetylation is broadly distributed and most highly detected next to the actual enhancer regions where the transcription factors are bound, methods that rely on histone acetylation alone for enhancer detection are inherently limited in resolution. In our approach, we alleviate this problem by independently gathering information on the likely position of enhancer regions (through CBP or ATAC-seq). However, enhancers are often in close proximity to each other; therefore, we cannot exclude the possibility that some of the identified regions show differential acetylation due to their proximity to bona fide enhancers (spill-over effect).

Indeed, we often identified multiple putative enhancers next to each other. These closely spaced putative enhancers could all be functional, since it is common for multiple enhancers to regulate a single gene’s expression pattern together [19, 44]. However, they may also include false positives due to the spill-over effect. Our validation assays, testing for proximity of putative enhancers to regulated genes and the 2 kb-long transgenic reporters, did not provide sufficient resolution to probe this potential source of false positives. A large fraction of false positives is unlikely though, since the identified regions were significantly enriched for the expected transcription factor motifs.

While the number of false positives in our DV enhancer list is unknown, there were false negatives, since our list did not include many of the known DV enhancers. We found that several known enhancers missed the cutoff for significance, not due to lack of differences in H3K27ac but due to noise. This suggests that future improvements in technology, such as tighter selection for embryos of the right stage and improved sequence coverage, have the potential to further improve the accuracy of our method. It will also be interesting to test whether adding data on the acetylation of other histone residues, such as H3K9ac or H3K16ac, H3K122ac, or H3K56ac [64], improves the results.

The advantage of differential acetylation analysis in combination with candidate region detection is that it does not require prior knowledge of the specific transcription factors involved in each tissue. Since histone modifications are well conserved across species and their antibody specificities are well characterized, this method should be broadly applicable to many uncharacterized developmental systems. The numbers of cells required as starting material for ChIP-seq are also lower for histone modifications than for transcription factors (and are even lower for ATAC-seq [32]). While the material requirements might still exceed what can be obtained from mammalian embryonic tissues, our method could be applied to mammalian in vitro differentiation systems, e.g., to detect differential enhancer activity in response to extracellular signals.

## Conclusions

Differential acetylation analysis provides an excellent starting point for identifying enhancers and their transcription factor binding motifs in an uncharacterized developmental regulatory network. The challenge in the future will be to make the generation of such data technically possible with relatively low amounts of starting material, e.g., when working with mammalian embryos. Undoubtedly, future improvements and innovations in genomics technology will greatly facilitate this goal.

## Methods

### Stock maintenance and embryo collection

The fly stock *Tl*^*10b*^ is from Indiana University (30914, Bloomington Stock Center, Bloomington, IN). The *gd*^*7*^ and *Tl*^*rm9/rm10*^ stocks were kind gifts from Mike Levine (Princeton University). For *Tl*^*10b*^ embryo collections, *T(1;3)OR60/Tl*^*10b*^*, e*^*1*^ females and *Tl*^*10b*^*/TM3, e*^*1*^*, Sb*^*1*^*, Ser*^*1*^ males were selected from the stock consisting of genotypes *Tl*^*10b*^*/TM3, e*^*1*^*, Sb*^*1*^*, Ser*^*1*^ and *T(1;3)OR60/TM3, e*^*1*^*, Sb*^*1*^*, Ser*^*1*^. The *gd*^*7*^*/gd*^*7*^ females and *gd*^*7*^/Y males were used for embryo collections and were obtained from the *gd*^*7*^*/winscy, hs-hid* stock by heat shocking 1-day-old larvae for 1 h at 37 °C, followed by a second heat shock 24 h later. *Tl*^*rm9/rm10*^ females and males were selected for embryo collections from offspring originating from the crossing of virgin females from the stock *Tl*^*rm9*^*/TM3, e*^*1*^*, Sb*^*1*^*, Ser*^*1*^ with males from the stock *Tl*^*rm10*^*/TM3, e*^*1*^*, Sb*^*1*^*, Ser*^*1*^. Oregon R embryos were used for wild-type samples.

All genotypes were expanded into population cages, and embryos were collected 2–4 h after egg deposition (AED). Apple juice plates were placed into the population cage at 25 °C for 2 h and then outside at 25 °C for another 2 h. For ChIP-seq experiments, embryos were dechorionated for 1 min with 100 % bleach and then cross-linked for 15 min with 1.8 % formaldehyde (final concentration in water phase). For mRNA-seq and ATAC-seq experiments, embryos were dechorionated but not cross-linked.

### ChIP-seq experiments

ChIP-seq experiments were performed as described [21, 23] with the following differences: ~100 mg embryos were used per ChIP and more extensive RIPA buffer washes were performed after H3K27ac ChIP incubation to reduce background. The antibodies used for ChIP-seq were custom-generated by GenScript: Dl (aa 39–346), Mad (aa 148–455), Zen (full length), Sna (full length), CBP (aa 2528–2872), and Zld (aa 1117–1327). Antibodies for Twi (aa 340–490) were custom-generated by Covance and antibodies for H3K27ac were obtained from Active motif, 39133. *Tl*^*10b*^ embryos were used for ChIP-seq for Dl and Twi, and H3K27ac, wild-type embryos for CBP and Sna, and *gd*^*7*^ embryos for Mad, Zen, Zld, and H3K27ac.

### Library preparation

Different combinations of library preparation kits and barcodes were used for library preparations (see Additional file 6: Table S4), and libraries were prepared according to manufacturer instructions. ChIP-seq libraries were prepared from 5–15 ng ChIP DNA or 100 ng input DNA and sequenced on the GAIIX (Illumina) or the HiSeq 2500 (Illumina).

### ChIP-seq data processing

All ChIP-seq samples were aligned to the *Drosophila melanogaster* UCSC dm3 reference genome using Bowtie v1.1.1 [65] with a maximum of two mismatches. Only uniquely aligning reads were used. The Bioconductor package chipseq was used to extend each sample’s aligned reads to the estimated fragment size. All ChIP-seq enrichment values were calculated as fold change over the corresponding input sample, after normalizing for differences in read count and fragment size. CBP peaks were first called with peakzilla [66] using default parameters for the CBP wild-type ChIP-seq and its corresponding input control. Detected peaks were resized to 201 bp centered at the summit, and those with less than twofold enrichment were excluded. To assess sample quality, MACS2 was run on all samples with their corresponding tissue’s input control and these non-default parameters:

- macs2 callpeak -t ip.bam -c wce.bam -g dm --keep-dup = all

Peak counts for each sample can be found in Additional file 6: Table S4.

### mRNA-seq experiments

Total mRNA was extracted from 20–100 mg *Tl*^*10b*^ embryos in duplicates and from *gd*^*7*^ and *Tl*^*rm9/rm10*^ embryos in triplicates using the Maxwell Total mRNA purification kit (AS1225, Promega) according to the manufacturer’s instructions. PolyA-mRNA was isolated using Dynabeads oligo (dT) (61002, Life Technologies). Libraries were prepared following the instructions of the TruSeq DNA Sample Preparation Kit (FC-121-2001, Illumina) and sequenced on the HiSeq 2500 (Illumina).

### mRNA-seq data processing

The mRNA-seq replicates from *Tl*^*rm9/rm10*^, *Tl*^*10b*^, and *gd*^*7*^ were aligned with TopHat v2.0.14 [67] to the FlyBase r5.57 reference genome and gene annotations with the following non-default parameters:

- tophat -G fb557_genes.gtf -I 20 -I 5000 --no-coverage-search --segment-length 25

Cuffdiff, from Cufflinks v2.2.1 [68], was used to determine transcript abundance and differential expression between all three pairwise combinations of DV mutants.

### ATAC-seq experiments

ATAC-seq was performed in biological duplicates using 20 mg 2–4 h AED Oregon R embryos. Nuclei were isolated by douncing the embryos in HBS buffer (0.125 M sucrose, 15 mM Tris (pH 7.5), 15 mM NaCl, 40 mM KCl, 2 mM EDTA, 0.5 mM EGTA) in a 2-ml dounce tissue grinder followed by filtering the nuclei suspension through Miracloth (475855, Calbiochem). Nuclei were spun at 500 *g* for 5 min at 4 °C, and the supernatant was discarded. ATAC-seq was performed as described [32] using 2.5 μl Tn*5* transposase and PCR reagents from the Nextera DNA Sample Preparation Kit (FC-121-1030, Illumina). In addition, the Nextera index kit (FC-121-1011, Illumina) was used to create libraries. Libraries were purified using Agencourt AMPure XP beads (A63881, Beckman Coulter), and paired-end sequencing was performed on the NextSeq (Illumina).

### ATAC-seq data processing

ATAC-seq paired-end reads were trimmed to 25 base pairs and aligned in paired-end mode with Bowtie v1.1.1, keeping only unique alignments with a maximum of two mismatches per read and an insert size of less than or equal to 1000 bp. MACS2 v2.1.0.20150420 [69] was used to identify ATAC peaks after combining both replicates using the following parameters:

- macs2 callpeak -t orer_combined_atac.bam -g dm -n orer_combined_atac -f BAMPE --call-summits

### Identification of differential H3K27ac regions

The Bioconductor package DESeq2 v1.10.1 [43] was used to identify peaks with differential H3K27ac read counts between the two or three replicates using default parameters and an adjusted *p* value cutoff of 0.01. For this, the reads overlapping a 1-kb window centered on the summit of either 201-bp CBP or ATAC reads were counted in two replicates in *gd*^*7*^ and three replicates in *Tl*^*10b*^. Regions were classified into TSS (less than 1000 bp from a FlyBase 5.57 annotated TSS), intragenic (inside an annotated gene but at least 1000 bp from a TSS), and intergenic (outside an annotated gene but at least 1000 bp from a TSS) enhancers. The average DESeq2 normalized ChIP signal for all replicates is shown in Fig. 1c and Additional file 3: Figure S3a.

### Metapeak profiles of putative DV enhancers

The average enrichment of *Tl*^*10b*^ H3K27ac, *gd*^*7*^ H3K27ac, and wild-type CBP ChIP-seq profiles over their respective input controls was calculated for putative MEs and DEEs after normalizing for differences in read count and fragment size. Enrichment values at each base relative to the CBP summit were smoothed with a 31-bp sliding window for display. ATAC-seq metapeak profiles display ATAC-seq average reads per million without any smoothing.

### Calculation of transcription factor enrichments

Transcription factor enrichments within our 201-bp putative enhancers were calculated by finding the summit within each enhancer for each transcription factor ChIP-seq sample and calculating the read count and fragment length-normalized enrichment over input in a 201-bp window around the summit.

### Nearest gene analysis

To assign regions (such as putative enhancers, non-differential regions, and transcription factor peaks) to the nearest gene, only genes with an mRNA-seq fragments per kilobase of exon per million fragments mapped (FPKM) of at least 5 in either *gd*^*7*^or *Tl*^*10b*^ were considered. Regions overlapping an expressed gene were assigned to that gene; otherwise, the gene with the closest TSS was assigned. For transcription factors, we selected the top 400 peaks by peakzilla score from the replicate with the most number of detected peaks.

### Known motif enrichment analysis

*Drosophila melanogaster* motifs in the Bioconductor MotifDb package v1.10 [70] were first filtered to include only those where the corresponding transcription factor is expressed in either *gd*^*7*^ or *Tl*^*10b*^ (mRNA-seq FPKM greater than 3). Motifs derived from *daughterless (da)* heterodimers in the FlyFactorSurvey database [45] were also excluded. The genomic locations of all remaining motifs were obtained using FIMO [47], part of the MEME Suite v4.10.1 [47], with the following parameters:

- fimo --text --bgfile dm3_background.markov motif.meme dm3.fasta

The background frequency file was obtained using the MEME tool fasta-get-markov against the modENCODE cold/warm/hot transcription factor binding regions (Dataset S8 from Roy et al. [71]).

Using a test and control group of regions, each region is scored for the presence or absence of every motif. The counts of regions containing the motif in the test group are compared to the counts of regions containing the motif in the control group using a one-sided proportion test. Resulting *p* values are corrected for multiple testing using the Benjamini-Hochberg method. As shown in Fig. 3a, putative MEs, DEEs, and non-differential regions were all 201 bp in width (centered at the CBP binding summit).

Additional file 3: Supplementary figures: Figure S2 shows the top 500 non-TSS peaks for each factor compared to all remaining non-TSS peaks. All regions were resized to 201 bp centered at the peak summit.

To combine similar motifs, the percentage of overlap among all occurrences was calculated between all pairs of significantly enriched motifs within either the putative enhancers (Fig. 3a) or all non-TSS peaks among the displayed factors (Additional file 3: Figure S2 and Figure S5). Motifs that overlapped each other in more than 10 % of occurrences were grouped as similar, and the motif with the lowest *p* value in the enrichment test was displayed as representative of the group.

### Motif conservation analysis

Per-base phastCons scores for the *Drosophila melanogaster* dm3 genome were downloaded from the UCSC Genome Browser (http://hgdownload.soe.ucsc.edu/goldenPath/dm3/database/phastCons15way) [72]. For each motif, the average phastCons score of all motif instances within each 201-bp putative DV enhancer was paired with the average phastCons score of the entire enhancer region. Significance was determined using a Wilcoxon paired rank-sum test. In addition, the average phastCons scores of all putative DV enhancers were compared to 5000 random non-TSS regions of equal width using a Wilcoxon unpaired rank-sum test.

### Transcription factor binding and motif presence analysis

As shown in Additional file 3: Figure S7, a two-sided Wilcoxon test is used to compare the ChIP-seq enrichment values of each transcription factor at putative DV enhancers containing each motif to those enhancers lacking the motif.

### Vienna Tiles analysis

As shown in Fig. 2d, annotated Vienna Tiles (VTs) overlapped by each region group (putative DV enhancers and the top 400 non-TSS peaks from Dl, Twi, Mad, and Zen ChIP-seq) were identified. The proportions of overlapping VTs in each group with annotated expression terms containing “mesoderm” and “amnioserosa” were determined and compared to all VTs overlapping non-differential CBP peaks using a one-sided proportion test.

A similar analysis was performed (see Fig. 2c) using the proportion of VTs with expression in stages 4–6, 7–8, or 9–10 compared to all annotated VTs.

### Transcription factor binding at selected putative DV enhancers

As shown in Fig. 5a, putative DV enhancers were selected based on the expression of the nearest gene and transcription factor binding. First, putative DV enhancers overlapping known enhancers were removed. Next, putative DV enhancers where the nearest gene’s mRNA-seq expression was highest in *Tl*^*rm9/rm10*^ compared to both *Tl*^*10b*^ and *gd*^*7*^ were also removed in order to exclude neuroectodermal genes. Finally, putative DV enhancers where the tissue of differential H3K27ac did not match the tissue of differential expression of the nearest gene were removed. For the remaining putative MEs, those with Dl ChIP-seq enrichment of at least threefold over input were assigned to the “Dl binding” group. Those with Twi ChIP-seq enrichment of at least fivefold but Dl ChIP-seq enrichment less than threefold were assigned to the “Twi” group. For the remaining putative DEEs, those with a Mad ChIP-seq enrichment of at least threefold were assigned to the “Mad binding” group, and those with a Zen ChIP-seq enrichment of at least threefold but a Mad ChIP-seq enrichment less than threefold were assigned to the “Zen binding” group. All other putative enhancers not assigned to one of the four transcription factor groups are not displayed. Enrichment values displayed in the heatmap were independently normalized for each factor to be between 0 (no enrichment over input or less) and 1 (98^th^ percentile enrichment or higher).

## Additional files

Additional file 1: Supplementary material: Includes references for known DV enhancers, ChIP-seq and ATAC-seq replicate correlations, and an overview of how some known DV enhancers were assigned to potential target genes. (DOCX 3548 kb)

Additional file 2: Table S1. Spreadsheet showing known DV enhancers assembled from the literature that were used in this study. (XLSX 14 kb)

Additional file 3: Supplementary figures: **Fig. S1**: Gene names for the data shown in Fig. 1a–c, **Fig. S2**: Transcription factor motifs enriched in top ChIP-seq and ATAC-seq regions, **Fig. S3**: Differential H3K27ac analysis of ATAC-seq regions is an effective method to identify tissue-specific enhancers, **Fig. S4**: Genes near putative ATAC-seq derived enhancers are differentially regulated across tissues, **Fig. S5**: The identified putative DV enhancer regions derived from ATAC-seq are enriched for known DV transcription factor motifs, **Fig. S6**: Number of genes with one or multiple assigned enhancers, **Fig. S7**: Transcription factor ChIP-seq signal is preferentially found at the expected corresponding binding motifs present within putative MEs and DEEs. (PDF 2673 kb)

Additional file 4: Table S2. Spreadsheet of all distal identified DV enhancers, the assigned gene and its expression in the DV mutants, H3K27ac and transcription factor ChIP enrichment, overlap with known DV enhancers and Vienna Tiles, enrichment for transcription factor motifs, and classification as high confidence enhancers (used in Fig.5). (XLSX 256 kb)

Additional file 5: Table S3. Spreadsheet showing all identified DV enhancers that overlap a gene’s TSS, the assigned gene and its expression in the DV mutants, H3K27ac and transcription factor ChIP enrichment, and overlap with known DV enhancers and Vienna Tiles. (XLSX 147 kb)

Additional file 6: Table S4. Spreadsheet detailing the samples used in this study and the library preparation kits the libraries were created with, number of total reads, aligned reads, and MACS peaks for each sample. (XLSX 11 kb)
