# Supplementary material for: Genome-wide identification of Drosophila dorso-ventral enhancers by differential histone acetylation analysis
Source: Genome Biol. 2016 Sep 27;17:196. doi: 10.1186/s13059-016-1057-2 (PMC5037609; doi:10.1186/s13059-016-1057-2)
Supplement: Supplementary file 1 — Supplementary material: Includes references for known DV enhancers, ChIP-seq and ATAC-seq replicate correlations, and an overview of how some known DV enhancers were assigned to potential target genes. (DOCX 3548 kb) [file 13059_2016_1057_MOESM1_ESM.docx]

**Supplementary material**

**Genome-wide identification of *Drosophila* dorso-ventral enhancers by differential histone acetylation analysis**

# References for known DV enhancers

| **Enhancer name** | **Coordinates (dm3)** | **Reference** |
| --- | --- | --- |
| Ady43A_Markstein | chr2R:3133869-3134085 | [Markstein et al. [1]](#_ENREF_1) |
| Ance_Rusch | chr2L:13904733-13905265 | [Rusch and Levine [2]](#_ENREF_2) |
| apt_Ozdemir | chr2R:19460067-19462066 | [Ozdemir et al. [3]](#_ENREF_3) |
| Asph_Ozdemir | chr2R:11997142-11999642 | [Ozdemir et al. [3]](#_ENREF_3) |
| BobA_Ozdemir | chr3L:14945343-14947303 | [Ozdemir et al. [3]](#_ENREF_3) |
| C15_650_Lin | chr3R:17331786-17332437 | [Lin et al. [4]](#_ENREF_4) |
| cact_Sandmann | chr2L:16319411-16320423 | [Sandmann et al. [5]](#_ENREF_5) |
| CG4221_Sandmann | chr3R:11746513-11748343 | [Sandmann et al. [5]](#_ENREF_5) |
| CG8788_Sandmann | chr2R:5025946-5026574 | [Sandmann et al. [5]](#_ENREF_5) |
| Cnx99A_Ozdemir | chr3R:25134141-25135377 | [Ozdemir et al. [3]](#_ENREF_3) |
| Cyp310a1_Ozdemir | chr2L:18652293-18653292 | [Ozdemir et al. [3]](#_ENREF_3) |
| dpp_Huang | chr2L:2455866-2457725 | [Huang et al. [6]](#_ENREF_6) |
| Dscam_Ozdemir | chr2R:3268100-3268475 | [Ozdemir et al. [3]](#_ENREF_3) |
| Dtg_Hodar | chr3R:8473379-8473902 | [Hodar et al. [7]](#_ENREF_7) |
| emc_Ozdemir | chr3L:742059-743659 | [Ozdemir et al. [3]](#_ENREF_3) |
| htl_Stathopoulos | chr3R:13875600-13876391 | [Stathopoulos et al. [8]](#_ENREF_8) |
| Ilp4_Stathopoulos | chr3L:9797449-9797743 | [Stathopoulos et al. [9]](#_ENREF_9) |
| Mdr49_Zeitlinger | chr2R:8833934-8834294 | [Zeitlinger et al. [10]](#_ENREF_10) |
| Mef2_Nguyen | chr2R:5819214-5819493 | [Nguyen and Xu [11]](#_ENREF_11) |
| Mef2_Ozdemir | chr2R:5847634-5848273 | [Ozdemir et al. [3]](#_ENREF_3) |
| mir-1_Biemar | chr2L:20480577-20481741 | [Biemar et al. [12]](#_ENREF_12) |
| mir-1_Zeitlinger | chr2L:20475806-20477901 | [Zeitlinger et al. [10]](#_ENREF_10) |
| Ocho_Ozdemir | chr3L:14968341-14970091 | [Ozdemir et al. [3]](#_ENREF_3) |
| Phm_Markstein | chr2R:19875348-19875790 | [Markstein et al. [1]](#_ENREF_1) |
| pnr_Zeitlinger | chr3R:11854234-11854784 | [Zeitlinger et al. [10]](#_ENREF_10) |
| pnt_Ozdemir | chr3R:19169181-19170380 | [Ozdemir et al. [3]](#_ENREF_3) |
| sna_Ip | chr2L:15478144-15481082 | [Ip et al. [13]](#_ENREF_13) |
| sna-S_Perry | chr2L:15485484-15486749 | [Perry et al. [14]](#_ENREF_14) |
| stumps_Sandmann | chr3R:10414437-10416493 | [Sandmann et al. [5]](#_ENREF_5) |
| T48_Sandmann | chr3R:22707641-22709469 | [Sandmann et al. [5]](#_ENREF_5) |
| tin_Yin | chr3R:17205819-17205999 | [Yin et al. [15]](#_ENREF_15) |
| tld_Kirov | chr3R:20574728-20575524 | [Kirov et al. [16]](#_ENREF_16) |
| trbl_Sandmann | chr3L:20394270-20396658 | [Sandmann et al. [5]](#_ENREF_5) |
| tup_Zeitlinger | chr2L:18875141-18875548 | [Zeitlinger et al. [10]](#_ENREF_10) |
| twi_Jiang | chr2R:18932429-18933663 | [Jiang et al. [17]](#_ENREF_17) |
| twi_Ozdemir | chr2R:18936997-18938126 | [Ozdemir et al. [3]](#_ENREF_3) |
| VT0984_Kvon | chr2L:1958014-1960258 | [Kvon et al. [18]](#_ENREF_18) |
| VT1404_Kvon | chr2L:2752765-2754884 | [Kvon et al. [18]](#_ENREF_18) |
| VT15341_Kvon | chr2R:7779541-7781099 | [Kvon et al. [18]](#_ENREF_18) |
| VT17873_Kvon | chr2R:12671212-12673370 | [Kvon et al. [18]](#_ENREF_18) |
| VT20119_Kvon | chr2R:16988930-16989475 | [Kvon et al. [18]](#_ENREF_18) |
| VT21015_Kvon | chr2R:18689548-18691090 | [Kvon et al. [18]](#_ENREF_18) |
| VT26325_Kvon | chr3L:5976078-5978250 | [Kvon et al. [18]](#_ENREF_18) |
| VT34695_Kvon | chr3L:22175053-22177267 | [Kvon et al. [18]](#_ENREF_18) |
| VT36350_Kvon | chr3R:282570-284657 | [Kvon et al. [18]](#_ENREF_18) |
| VT37495_Kvon | chr3R:2548627-2550765 | [Kvon et al. [18]](#_ENREF_18) |
| VT40612_Kvon | chr3R:8473805-8476580 | [Kvon et al. [18]](#_ENREF_18) |
| VT42492_Kvon | chr3R:12076924-12079107 | [Kvon et al. [18]](#_ENREF_18) |
| VT44117_Kvon | chr3R:15157311-15159508 | [Kvon et al. [18]](#_ENREF_18) |
| VT44757_Kvon | chr3R:16368758-16370878 | [Kvon et al. [18]](#_ENREF_18) |
| VT49279_Kvon | chr3R:25088511-25090669 | [Kvon et al. [18]](#_ENREF_18) |
| VT56148_Kvon | chrX:3032372-3034558 | [Kvon et al. [18]](#_ENREF_18) |
| VT57022_Kvon | chrX:4833051-4835256 | [Kvon et al. [18]](#_ENREF_18) |
| VT6477_Kvon | chr2L:12665181-12667293 | [Kvon et al. [18]](#_ENREF_18) |
| VT7402_Kvon | chr2L:14485084-14487223 | [Kvon et al. [18]](#_ENREF_18) |
| VT8889_Kvon | chr2L:17381424-17383547 | [Kvon et al. [18]](#_ENREF_18) |
| VT9677_Kvon | chr2L:18893565-18895695 | [Kvon et al. [18]](#_ENREF_18) |
| wntD_Zeitlinger | chr3R:9118955-9119462 | [Zeitlinger et al. [10]](#_ENREF_10) |
| zen_dist_Doyle | chr3R:2580742-2581377 | [Doyle et al. [19]](#_ENREF_19) |
| zen_prox_Doyle | chr3R:2580211-2580586 | [Doyle et al. [19]](#_ENREF_19) |

# Consistency of ChIP-seq and ATAC-seq replicates

The consistency between ChIP-seq replicates was determined by calculating the Pearson correlation across 1000 bp bins covering the whole genome for all samples. Most replicates were performed on different days, often using different library preparation kits (see Additional file 5 Table S4).

| **Sample** | **Pearson correlation** |
| --- | --- |
| H3K27ac *gd^7^* | 0.9533 |
| Mad *gd^7^* | 0.9157 |
| Zen *gd^7^* | 0.971 |
| Zld *gd^7^* | 0.9372 |
| H3K27ac *Tl^10b^* | 0.9238 |
| Dl *Tl^10b^* | 0.6409 |
| Twi *Tl^10b^* | 0.8202 |
| ATAC-seq wt | 0.9824 |

# Assignment of some known DV enhancers to potential target genes

Enhancers from [Kvon et al. [18]](#_ENREF_18) and [Ozdemir et al. [3]](#_ENREF_3) were originally discovered independently of a target gene and thus their target genes are uncertain. For Fig. 1A,B and Fig. S1 in Additional file 3: Supplementary figures, enhancers were assigned to the most likely target gene based on the similarity of the gene’s expression domain to the expression domain driven by the enhancer in question. As likely target genes, we considered (1) the nearest or overlapping gene assigned by hand, or the gene to which the enhancer was previously assigned by [Ozdemir et al. [3]](#_ENREF_3) by similar means (‘Gene assigned by hand’), and (2) the nearest TSS with a reasonable amount of transcript levels (FPKM > 5) in *Tl^10b^* or *gd^7^* mRNA-seq (‘Nearest expressed gene’). When we considered genes further away, no additional target gene candidate was identified with confidence. The expression domains of the enhancers provided by [Kvon et al. [18]](#_ENREF_18) (Supplemental Fig. S2) and [Ozdemir et al. [3]](#_ENREF_3) (Sup Fig. 3) were compared with the expression domain of the putative target genes documented by the BDGP database (Berkeley *Drosophila* Genome Project) [[20-22](#_ENREF_20)] or published expression patterns, as indicated. If the gene’s and the enhancer’s expression domain were similar, the gene was assigned as the enhancer’s most likely target gene (see below and Additional file 2: Table S1). If the gene’s and the enhancer’s expression domain were not similar or if no *in situ* hybridization stainings for a gene’s expression domain could be found, the target gene was assigned as “Unknown” in Additional file 2: Table S1 (also see below) and these genes were not utilized for the analysis shown in Fig. 1 A,B and Fig. S1 in Additional file 3: Supplementary figures.

**Enhancers whose gene assigned by hand is the correct target gene:**

| **Enhancer name** | **Enhancer expression in** | **Most likely target gene** | **Gene assigned by hand** | **Nearest expressed gene** | **Gene expression from BDGP [**[**20-22**](#_ENREF_20)**]** | **Enhancer expression from** [**Ozdemir et al. [3]**](#_ENREF_3)**^1^ or** [**Kvon et al. [18]**](#_ENREF_18)**^2^ (VT…)** |
| --- | --- | --- | --- | --- | --- | --- |
| apt_Ozdemir | Mesoderm | **apt** | apt  FBgn0015903 | apt  FBgn0015903 | apt  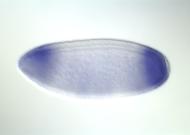 | apt_Ozdemir  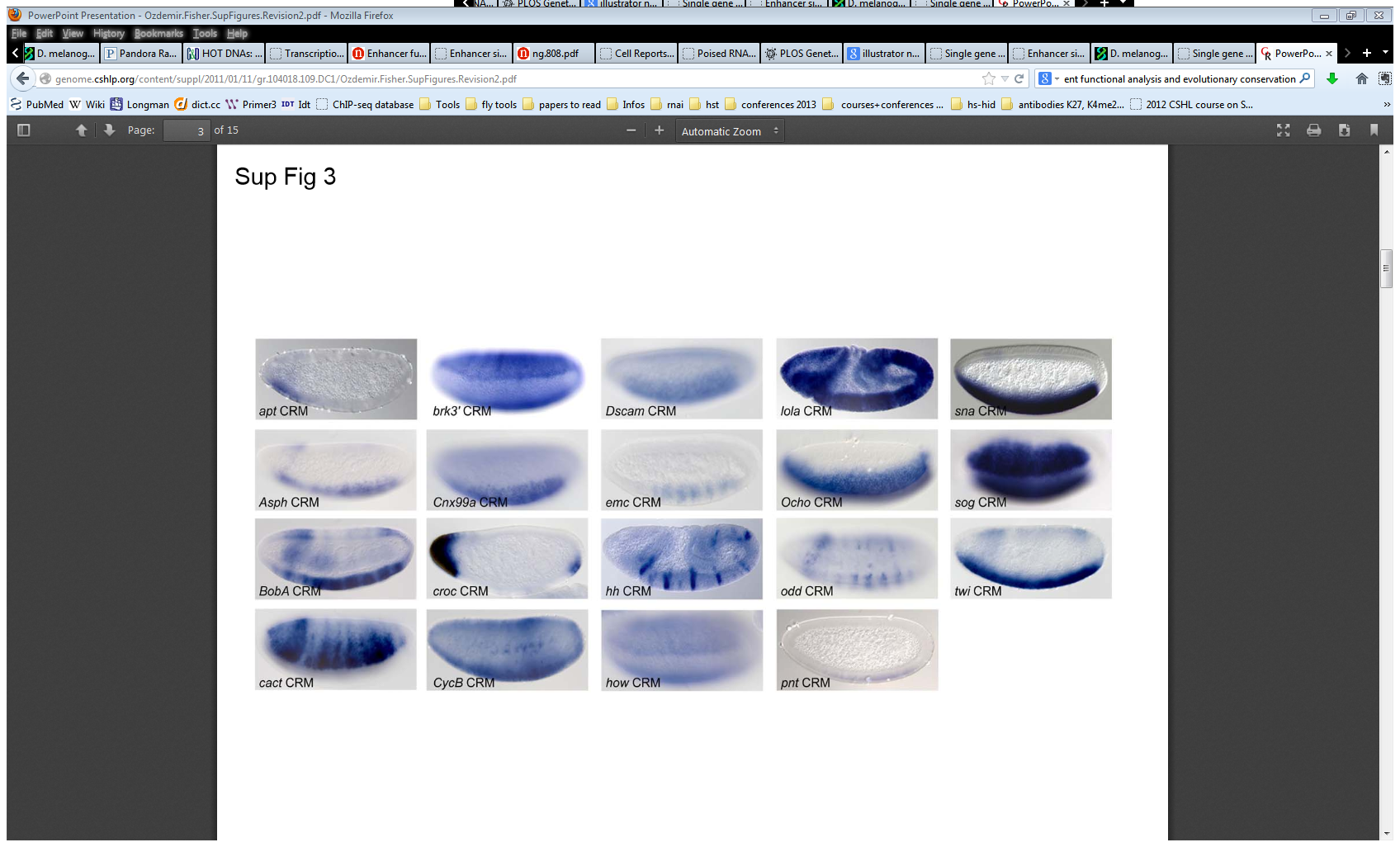 |
| Asph_Ozdemir | Mesoderm | **Asph** | Asph  FBgn0034075 | Ric  FBgn0265605 | Asph  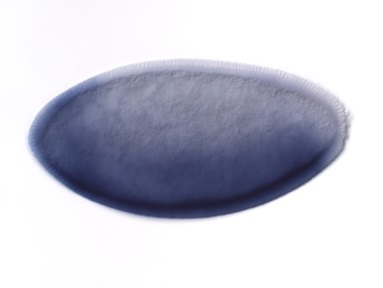  Ric  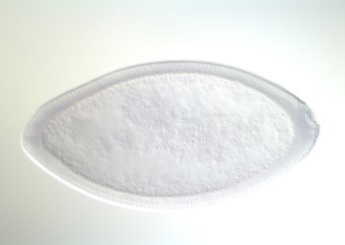 | Asph_Ozdemir  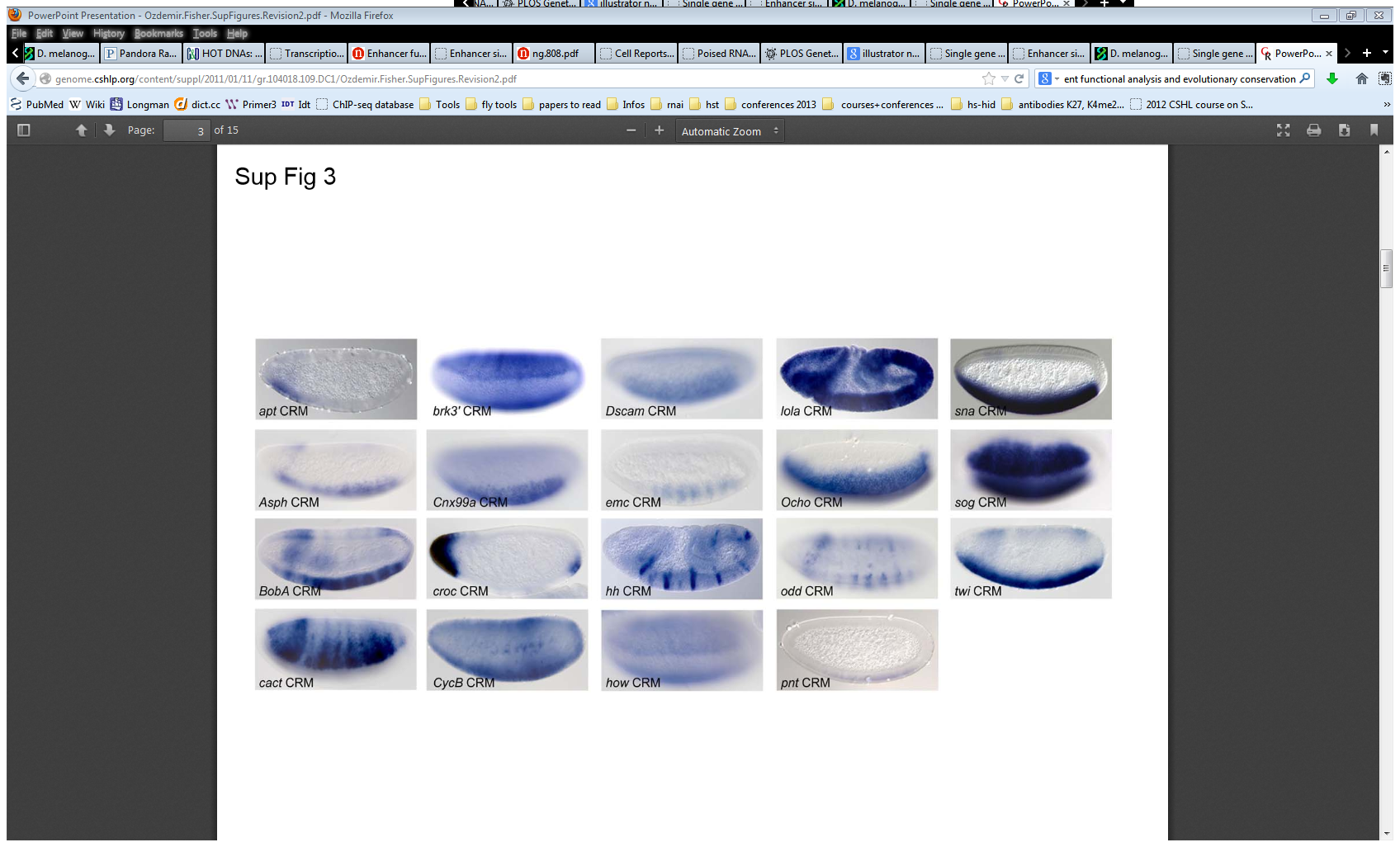 |
| Cyp310a1_Ozdemir | Mesoderm | **Cyp310a1** | Cyp310a1  FBgn0032693 | MESR3  FBgn0032694 | Cyp310a1  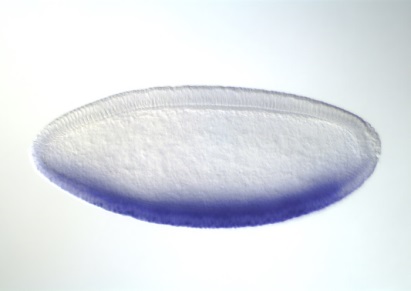  MESR3  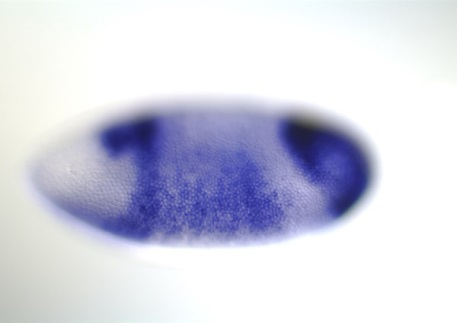 | Cyp310a1_Ozdemir  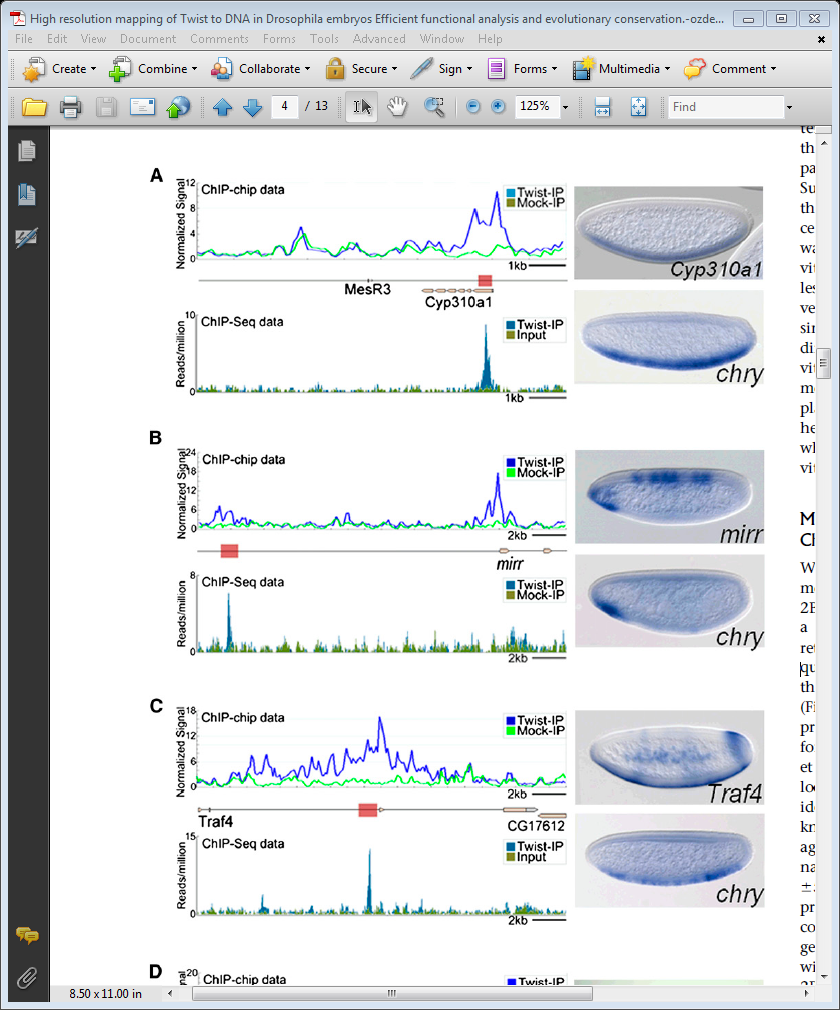 |
| Mef2_Ozdemir | Mesoderm | **Mef2** | Mef2  FBgn0011656 | Pal1  FBgn0033466 | Mef2  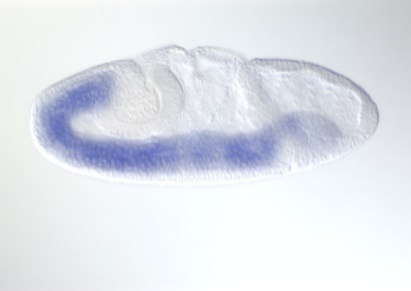  Pal1  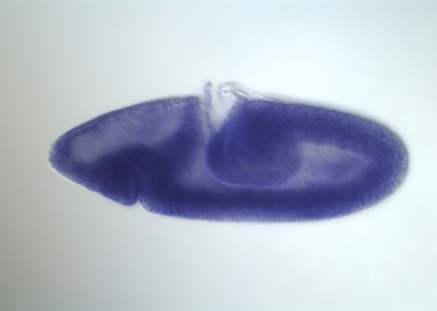 | Mef2_Ozdemir  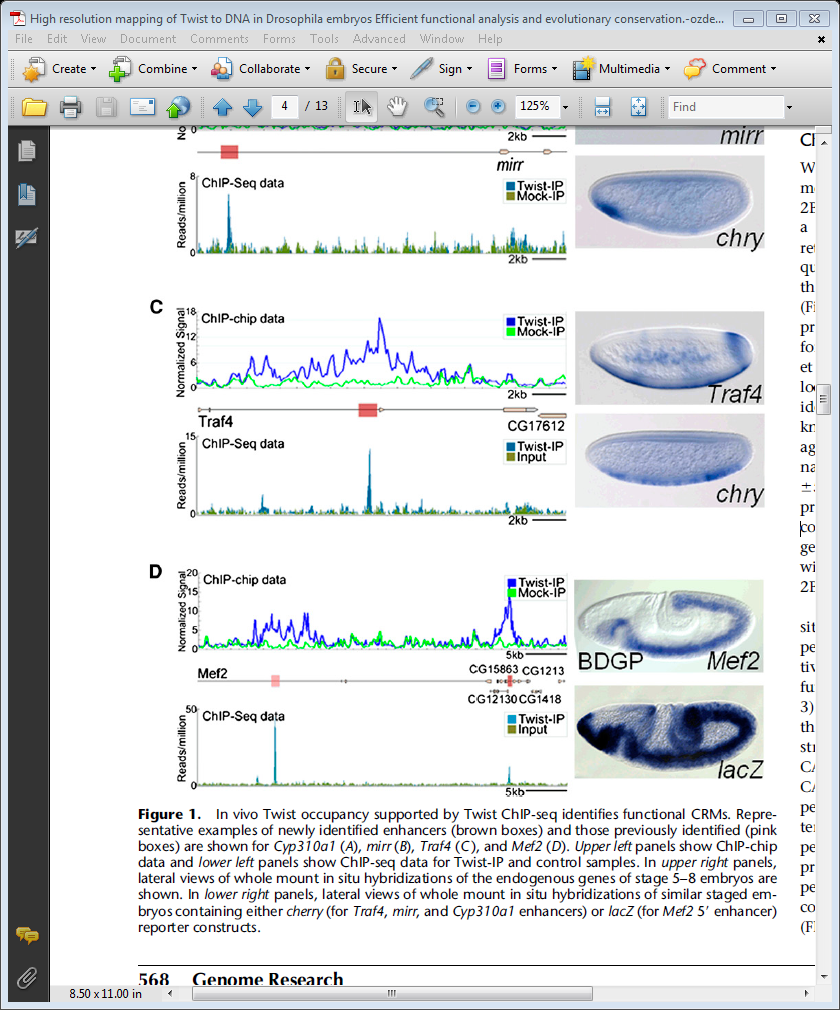 |
| twi_Ozdemir | Mesoderm | **twi** | twi  FBgn0003900 | twi  FBgn0003900 | twi  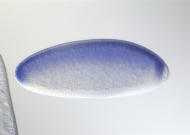 | twi_Ozdemir  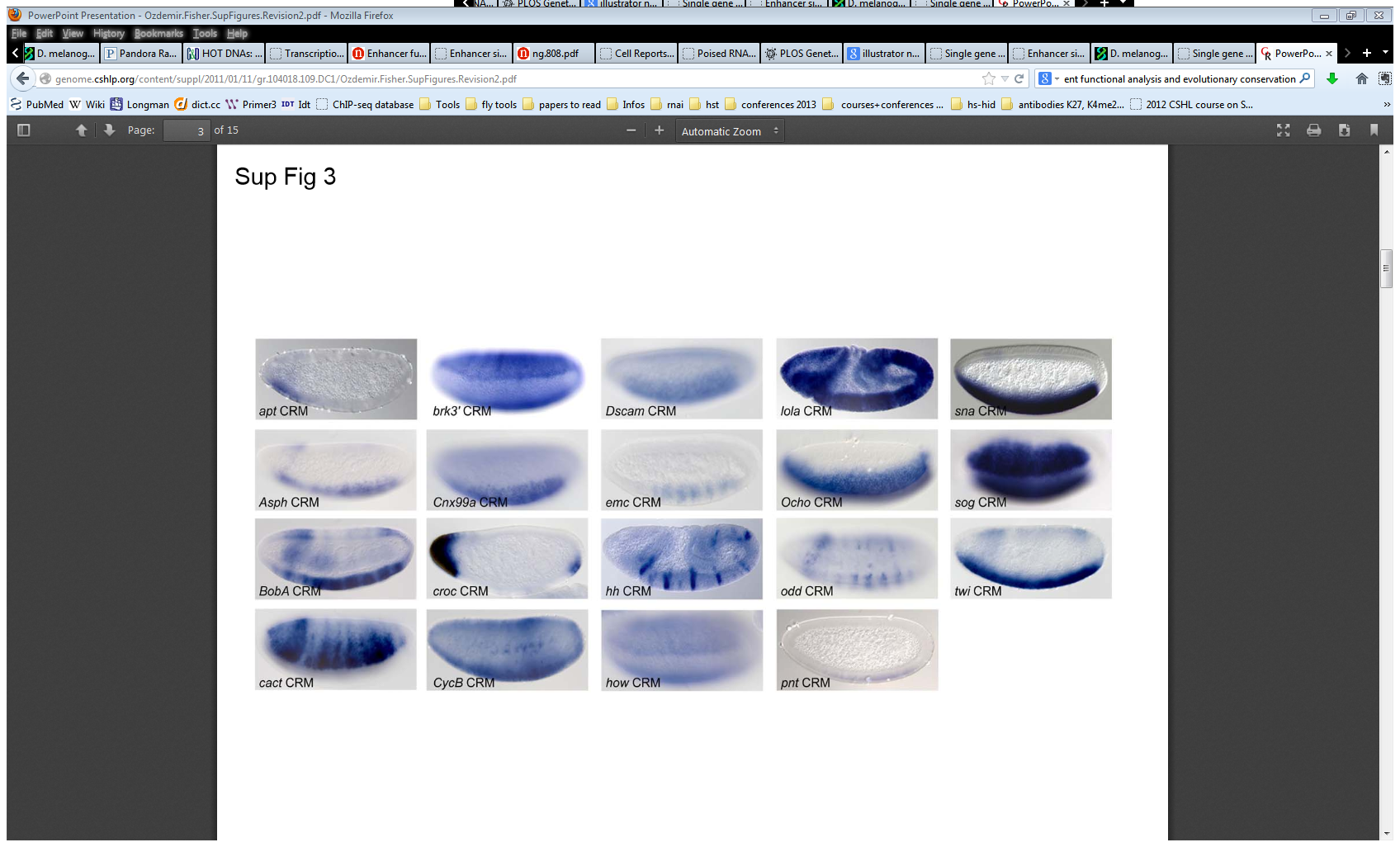 |
| VT0984_Kvon | Dorsal ectoderm | **erm** | erm  FBgn0031375 | Der-1  FBgn0031376 | erm  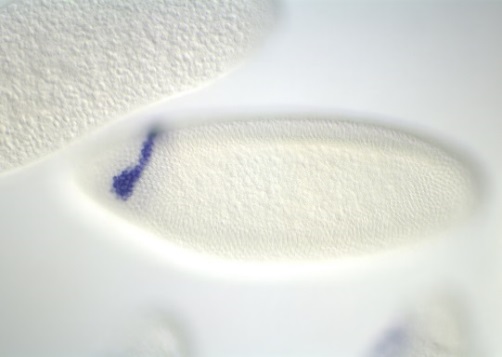  Der-1  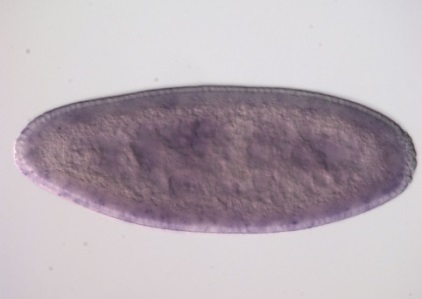 | 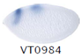 |
| VT17873_Kvon | Mesoderm | **CG5522** | CG5522  FBgn0034158 | CG5522  FBgn0034158 | CG5522  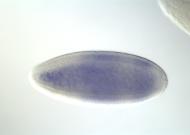 | 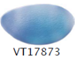 |
| VT20119_Kvon | Mesoderm | **ktub** | ktub  FBgn0015721 | ktub  FBgn0015721 | ktub  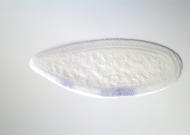 | 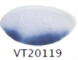 |
| VT26325_Kvon | Dorsal ectoderm | **CG10479** | CG10479  FBgn0035656 | CG10479  FBgn0035656 | CG10479  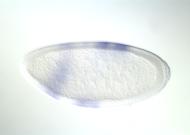 | 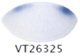 |
| VT37495_Kvon | Dorsal ectoderm | **zen2** | pb  FBgn0051481 | zen2  FBgn0004054 | pb  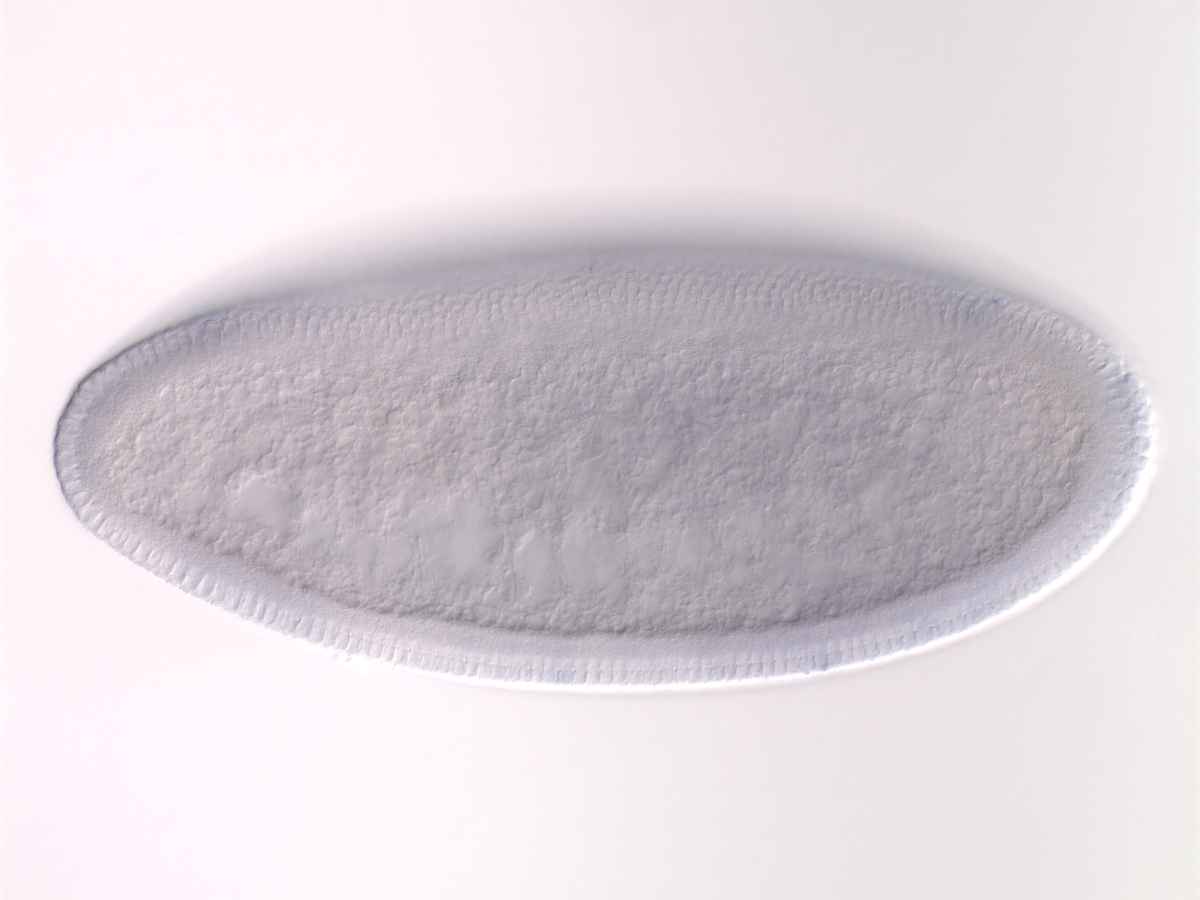  zen2  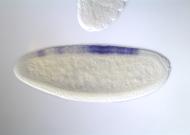 | 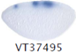 |
| VT42492_Kvon | Dorsal ectoderm | **tara** | tara  FBgn0040071 | tara  FBgn0040071 | tara  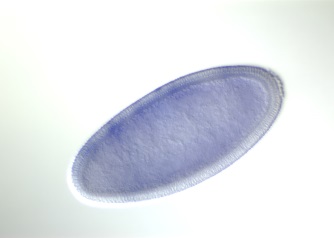 | 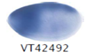 |
| VT44117_Kvon | Dorsal ectoderm | **Dl** | Dl  FBgn0000463 | Dl  FBgn0000463 | Dl  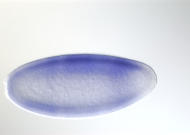 | 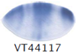 |
| VT56148_Kvon | Mesoderm | **N** | N  FBgn0004647 | N  FBgn0004647 | N  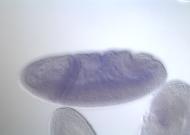 | 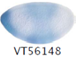 |
| VT6477_Kvon | Dorsal ectoderm | **pdm2** | pdm2  FBgn0004394 | pdm2  FBgn0004394 | pdm2  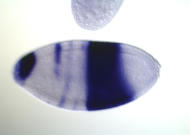 | 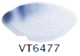 |
| VT7402_Kvon | Mesoderm | **noc** | noc  FBgn0005771 | noc  FBgn0005771 | noc  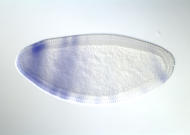 | 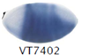 |
| VT9677_Kvon | Dorsal ectoderm | **tup** | tup  FBgn0003896 | tup  FBgn0003896 | tup  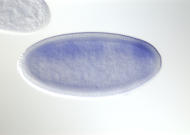 | 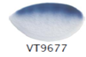 |

^1^ Copyright 2011 Cold Spring Harbor Laboratory Press

^2^ Copyright 2012 Cold Spring Harbor Laboratory Press

**Enhancers whose nearest expressed gene was assigned:**

| **Enhancer name** | **Enhancer expression in** | **Likely target gene** | **Gene assigned by hand** | **Nearest expressed**  **gene** | **Gene expression from BDGP [**[**20-22**](#_ENREF_20)**]** | **Enhancer expression from** [**Kvon et al. [18]**](#_ENREF_18)**^2^ (VT…)** |
| --- | --- | --- | --- | --- | --- | --- |
| VT8889_Kvon | Mesoderm | Lrch | CG15141  FBgn0032635 | Lrch  FBgn0032633 | Lrch  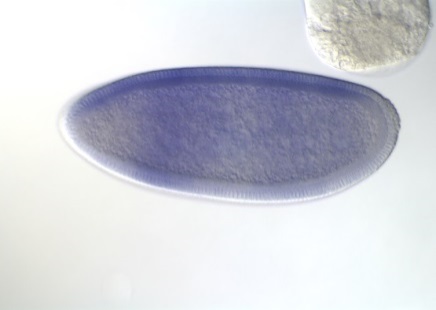  CG15141  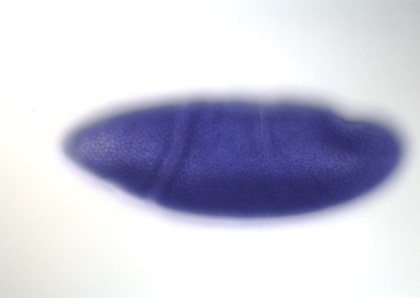 | 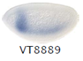 |

***Enhancers with unknown target gene:***

| **Enhancer name** | **Enhancer expression in** | **Likely target gene** | **Gene assigned by hand** | **Nearest expressed**  **gene** | **Gene expression from BDGP [**[**20-22**](#_ENREF_20)**] or publication** | **Enhancer expression from** [**Ozdemir et al. [3]**](#_ENREF_3)**^1^ or** [**Kvon et al. [18]**](#_ENREF_18)**^2^ (VT…)** |
| --- | --- | --- | --- | --- | --- | --- |
| BobA_Ozdemir | Mesoderm | **Unknown:**  **target gene not expressed in mesoderm** | BobA  FBgn0040487 | BobA  FBgn0040487 | BobA  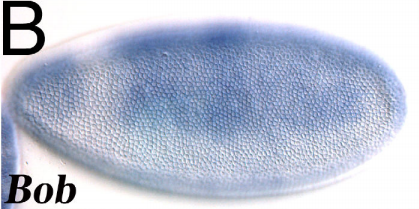  [Bardin and Schweisguth [23]](#_ENREF_23)^3^ | BobA_Ozdemir  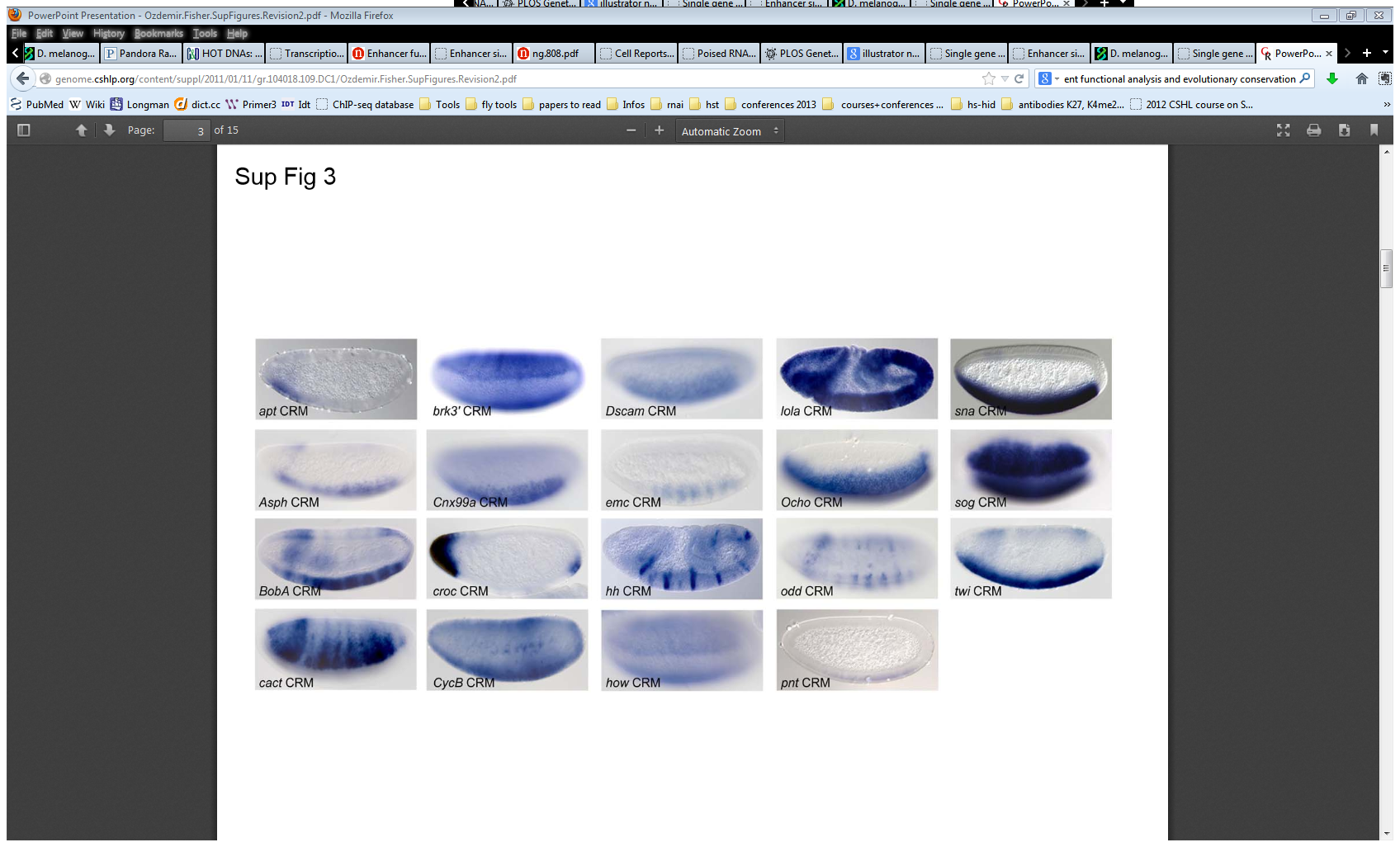 |
| Cnx99A_Ozdemir | Mesoderm | **Unknown: no early expression pattern found for target gene** | Cnx99A  FBgn0015622 | Cnx99A  FBgn0015622 | No early expression pattern found for Cnx99A | Cnx99A_Ozdemir  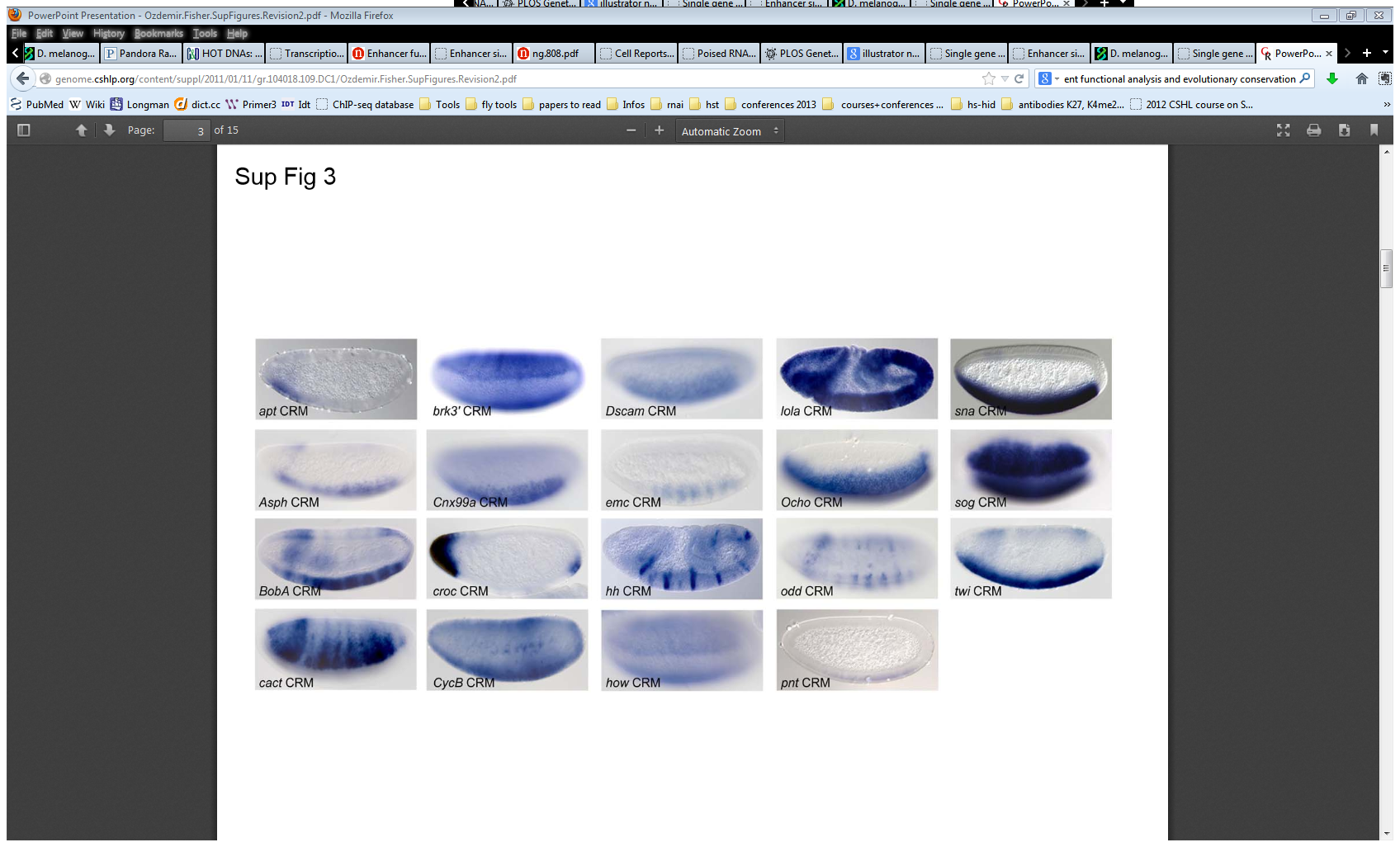 |
| Dscam1_Ozdemir | Mesoderm | **Unknown: target gene expression pattern not similar** | Dscam1  FBgn0033159 | Dscam1  FBgn0033159 | Dscam1  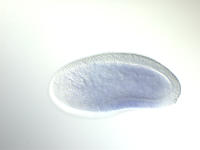 | Dscam_Ozdemir  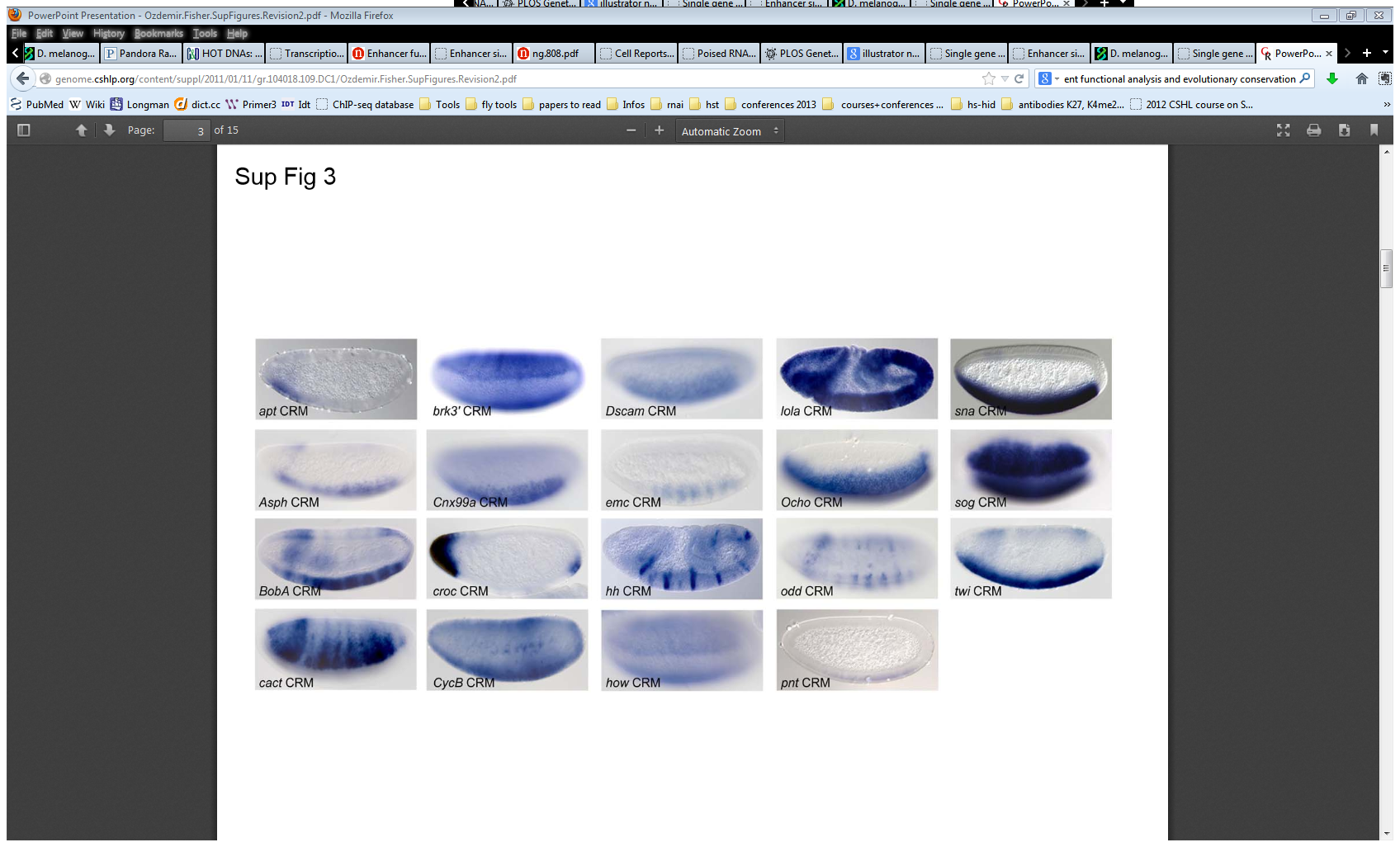 |
| emc_Ozdemir | Mesoderm | **Unknown: target genes are not expressed in mesoderm** | emc  FBgn0000575 | hng3  FBgn0035160 | emc  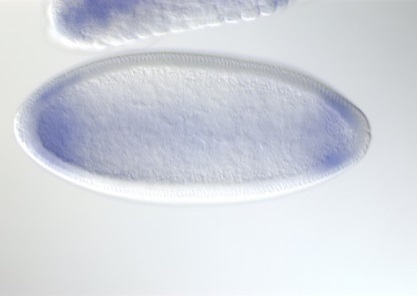  hng3  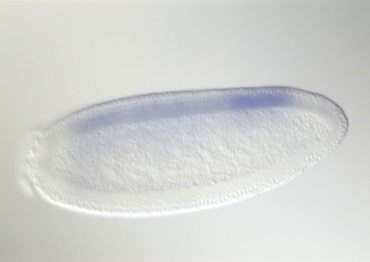 | emc_Ozdemir  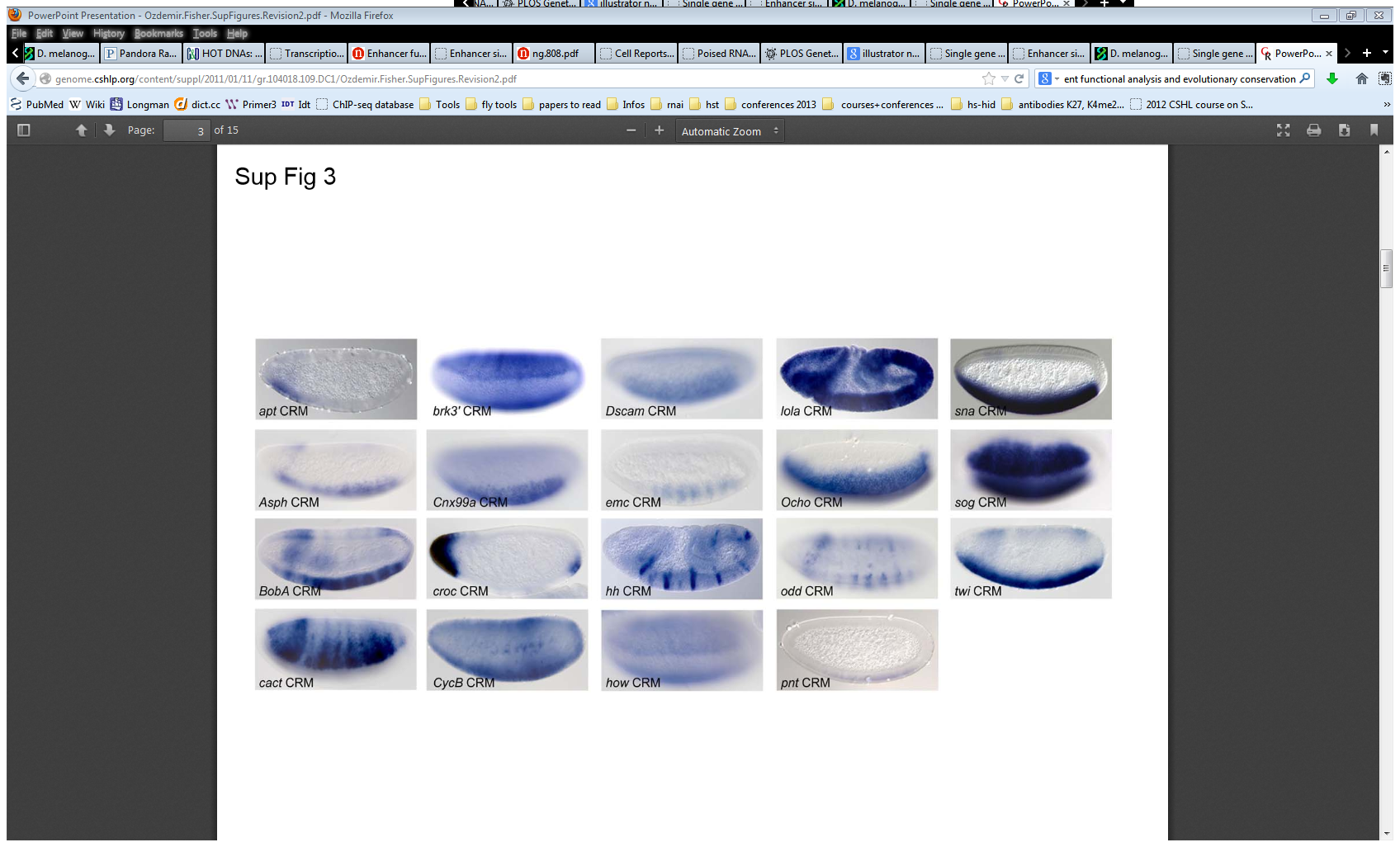 |
| Ocho_Ozdemir | Mesoderm | **Unknown: target gene expressed ubiquitously** | Ocho  FBgn0040296 | CR43432  FBgn0263380 | Ocho  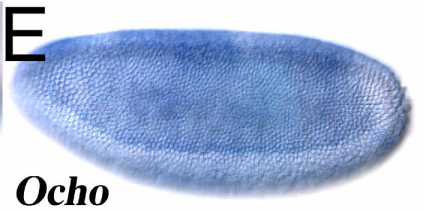  [Bardin and Schweisguth [23]](#_ENREF_23)^3^  No expression pattern found for CR43432 | Ocho_Ozdemir  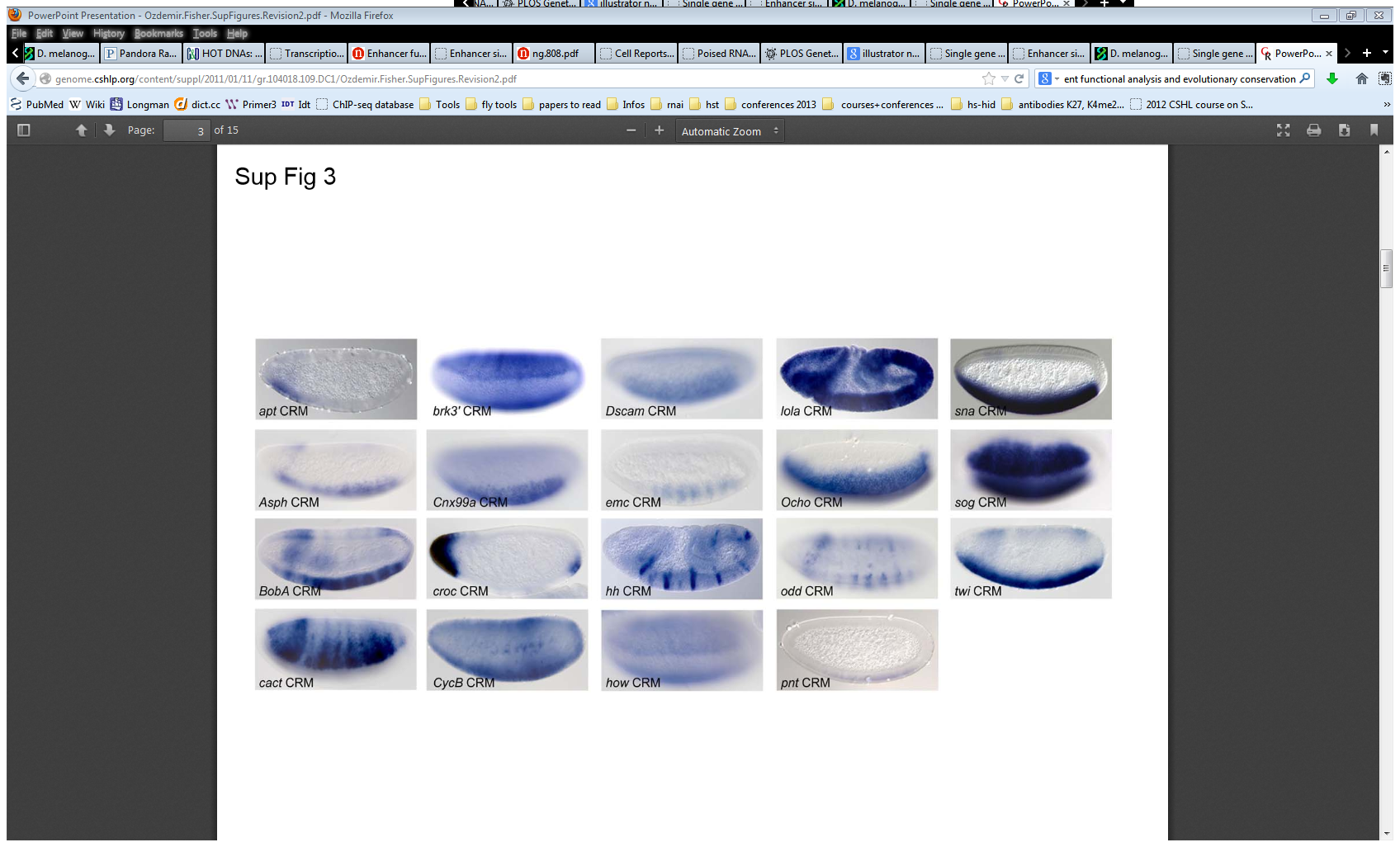 |
| pnt_Ozdemir | Mesoderm | **Unknown: target gene not expressed in mesoderm** | pnt  FBgn0003118 | pnt  FBgn0003118 | pnt  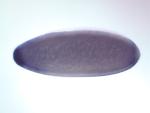 | pnt_Ozdemir  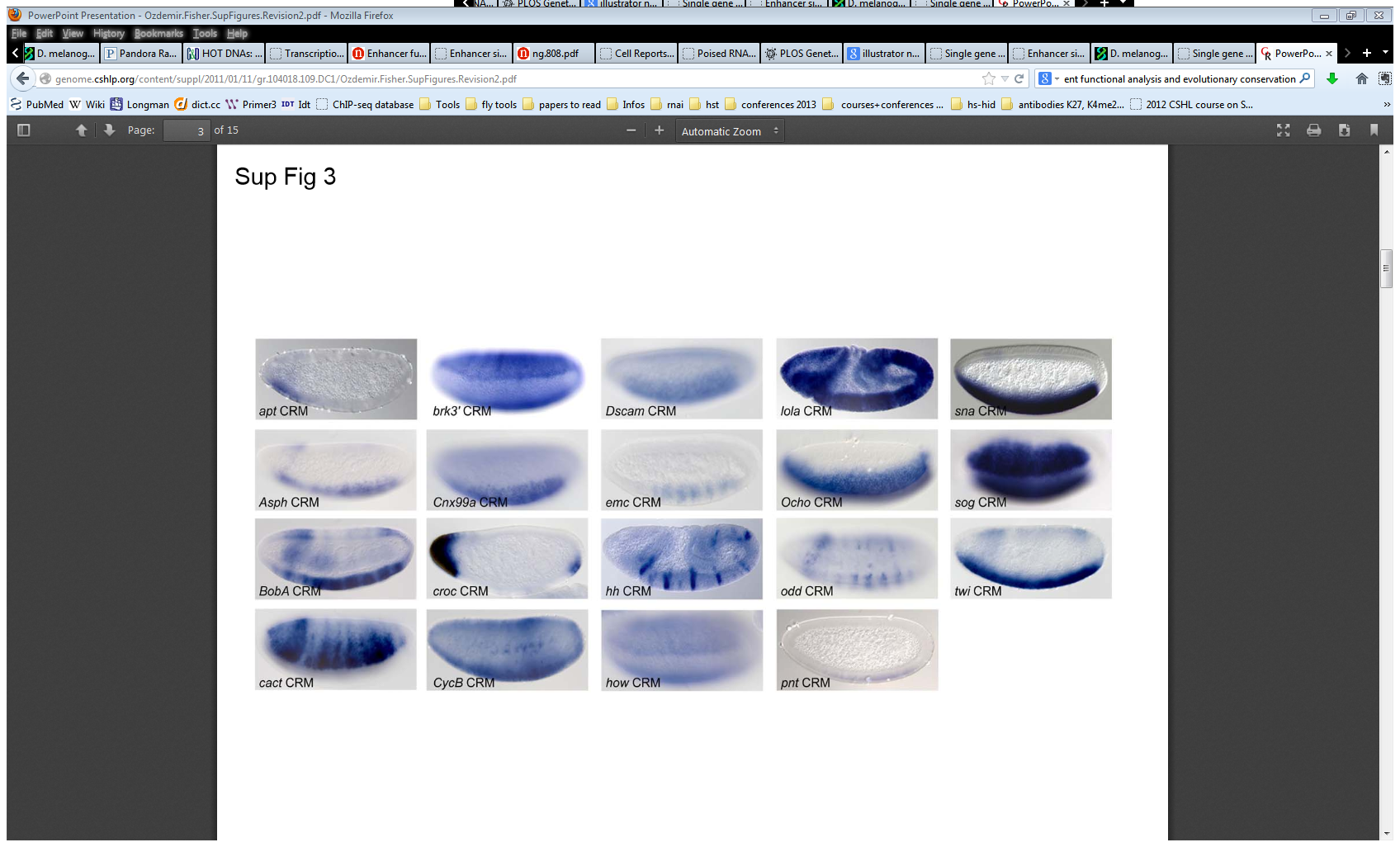 |
| VT1404_Kvon | Dorsal ectoderm | **Unknown: target gene does not show expression by in situ** | Pgk  FBgn0250906 | Pgk  FBgn0250906 | Pgk  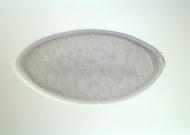 | 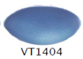 |
| VT15341_Kvon | Mesoderm | **Unknown: no expression pattern found for target gene** | snoRNA: Me28S-A1322  FBgn0015543 | snoRNA: Me28S-A1322  FBgn0015543 | No expression pattern found for snoRNA: Me28S-A1322 | 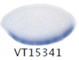 |
| VT21015_Kvon | Mesoderm | **Unknown: target gene expressed ubiquitously** | blw  FBgn0011211 | blw  FBgn0011211 | blw  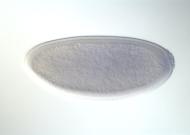 | 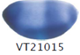 |
| VT34695_Kvon | Dorsal ectoderm | **Unknown: target gene not expressed or in situ not available** | olf413  FBgn0037153 | Rich  FBgn0028500 | olf413  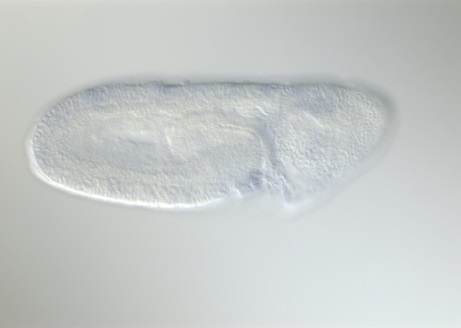  No expression pattern found for Rich | 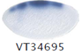 |
| VT36350_Kvon | Dorsal ectoderm | **Unknown: target gene expressed ubiquitously** | CG31522  FBgn0051522 | CG31522  FBgn0051522 | CG31522  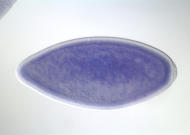 | 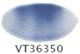 |
| VT44757_Kvon | Dorsal ectoderm | **Unknown: target gene expression pattern not similar** | Stat92E  FBgn0016917 | Stat92E  FBgn0016917 | Stat92E  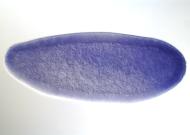 | 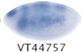 |
| VT49279_Kvon | Dorsal ectoderm | **Unknown: target gene not expressed in dorsal ectoderm** | stg  FBgn0003525 | stg  FBgn0003525 | stg  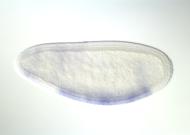 | 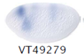 |
| VT57022_Kvon | Dorsal ectoderm | **Unknown: target gene expression pattern not similar** | Ptp4E  FBgn0004368 | Ptp4E  FBgn0004368 | Ptp4E  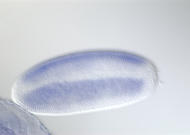 | 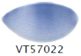 |

^3^ Reprinted from Cell, 10(2), Bardin and Schweisguth, Bearded Family Members Inhibit Neuralized-Mediated Endocytosis and Signaling Activity of Delta in Drosophila, 245-255, Copyright 2006, with permission from Elsevier.

# Supplemental references

1. Markstein M, Zinzen R, Markstein P, Yee KP, Erives A, Stathopoulos A, et al. A regulatory code for neurogenic gene expression in the Drosophila embryo*.* Development. 2004;131:2387-94.

2. Rusch J and Levine M. Regulation of a dpp target gene in the Drosophila embryo*.* Development. 1997;124:303-11.

3. Ozdemir A, Fisher-Aylor KI, Pepke S, Samanta M, Dunipace L, McCue K, et al. High resolution mapping of Twist to DNA in Drosophila embryos: Efficient functional analysis and evolutionary conservation*.* Genome Res. 2011;21:566-77.

4. Lin MC, Park J, Kirov N, and Rushlow C. Threshold response of C15 to the Dpp gradient in Drosophila is established by the cumulative effect of Smad and Zen activators and negative cues*.* Development. 2006;133:4805-13.

5. Sandmann T, Girardot C, Brehme M, Tongprasit W, Stolc V, and Furlong EE. A core transcriptional network for early mesoderm development in Drosophila melanogaster*.* Genes Dev. 2007;21:436-49.

6. Huang JD, Schwyter DH, Shirokawa JM, and Courey AJ. The interplay between multiple enhancer and silencer elements defines the pattern of decapentaplegic expression*.* Genes Dev. 1993;7:694-704.

7. Hodar C, Zuniga A, Pulgar R, Travisany D, Chacon C, Pino M, et al. Comparative gene expression analysis of Dtg, a novel target gene of Dpp signaling pathway in the early Drosophila melanogaster embryo*.* Gene. 2014;535:210-7.

8. Stathopoulos A, Tam B, Ronshaugen M, Frasch M, and Levine M. pyramus and thisbe: FGF genes that pattern the mesoderm of Drosophila embryos*.* Genes Dev. 2004;18:687-99.

9. Stathopoulos A, Van Drenth M, Erives A, Markstein M, and Levine M. Whole-genome analysis of dorsal-ventral patterning in the Drosophila embryo*.* Cell. 2002;111:687-701.

10. Zeitlinger J, Zinzen RP, Stark A, Kellis M, Zhang H, Young RA, et al. Whole-genome ChIP-chip analysis of Dorsal, Twist, and Snail suggests integration of diverse patterning processes in the Drosophila embryo*.* Genes Dev. 2007;21:385-90.

11. Nguyen HT and Xu X. Drosophila mef2 expression during mesoderm development is controlled by a complex array of cis-acting regulatory modules*.* Dev Biol. 1998;204:550-66.

12. Biemar F, Nix DA, Piel J, Peterson B, Ronshaugen M, Sementchenko V, et al. Comprehensive identification of Drosophila dorsal-ventral patterning genes using a whole-genome tiling array*.* Proc Natl Acad Sci U S A. 2006;103:12763-8.

13. Ip YT, Park RE, Kosman D, Yazdanbakhsh K, and Levine M. dorsal-twist interactions establish snail expression in the presumptive mesoderm of the Drosophila embryo*.* Genes Dev. 1992;6:1518-30.

14. Perry MW, Boettiger AN, Bothma JP, and Levine M. Shadow enhancers foster robustness of Drosophila gastrulation*.* Curr Biol. 2010;20:1562-7.

15. Yin Z, Xu XL, and Frasch M. Regulation of the twist target gene tinman by modular cis-regulatory elements during early mesoderm development*.* Development. 1997;124:4971-82.

16. Kirov N, Childs S, O'Connor M, and Rushlow C. The Drosophila dorsal morphogen represses the tolloid gene by interacting with a silencer element*.* Mol Cell Biol. 1994;14:713-22.

17. Jiang J, Kosman D, Ip YT, and Levine M. The dorsal morphogen gradient regulates the mesoderm determinant twist in early Drosophila embryos*.* Genes Dev. 1991;5:1881-91.

18. Kvon EZ, Stampfel G, Yanez-Cuna JO, Dickson BJ, and Stark A. HOT regions function as patterned developmental enhancers and have a distinct cis-regulatory signature*.* Genes Dev. 2012;26:908-13.

19. Doyle HJ, Kraut R, and Levine M. Spatial regulation of zerknullt: a dorsal-ventral patterning gene in Drosophila*.* Genes Dev. 1989;3:1518-33.

20. Hammonds AS, Bristow CA, Fisher WW, Weiszmann R, Wu S, Hartenstein V, et al. Spatial expression of transcription factors in Drosophila embryonic organ development*.* Genome Biol. 2013;14:R140.

21. Tomancak P, Beaton A, Weiszmann R, Kwan E, Shu S, Lewis SE, et al. Systematic determination of patterns of gene expression during Drosophila embryogenesis*.* Genome Biol. 2002;3:RESEARCH0088.

22. Tomancak P, Berman BP, Beaton A, Weiszmann R, Kwan E, Hartenstein V, et al. Global analysis of patterns of gene expression during Drosophila embryogenesis*.* Genome Biol. 2007;8:R145.

23. Bardin AJ and Schweisguth F. Bearded family members inhibit Neuralized-mediated endocytosis and signaling activity of Delta in Drosophila*.* Dev Cell. 2006;10:245-55.
