# Supplementary material for: Genome-wide identification of Drosophila dorso-ventral enhancers by differential histone acetylation analysis
Source: Genome Biol. 2016 Sep 27;17:196. doi: 10.1186/s13059-016-1057-2 (PMC5037609; doi:10.1186/s13059-016-1057-2)
Supplement: Supplementary file 3 — Supplementary figures: Fig. S1: Gene names for the data shown in Fig. 1a–c, Fig. S2: Transcription factor motifs enriched in top ChIP-seq and ATAC-seq regions, Fig. S3: Differential H3K27ac analysis of ATAC-seq regions is an effective method to identify tissue-specific enhancers, Fig. S4: Genes near putative ATAC-seq derived enhancers are differentially regulated across tissues, Fig. S5: The identified putative DV enhancer regions derived from ATAC-seq are enriched for known DV transcription factor motifs, Fig. S6: Number of genes with one or multiple assigned enhancers, Fig. S7: Transcription factor ChIP-seq signal is preferentially found at the expected corresponding binding motifs present within putative MEs and DEEs. (PDF 2673 kb) [file 13059_2016_1057_MOESM3_ESM.pdf]

Supplementary figures

Genome-wide identification of *Drosophila* dorso-ventral enhancers by differential histone acetylation analysis

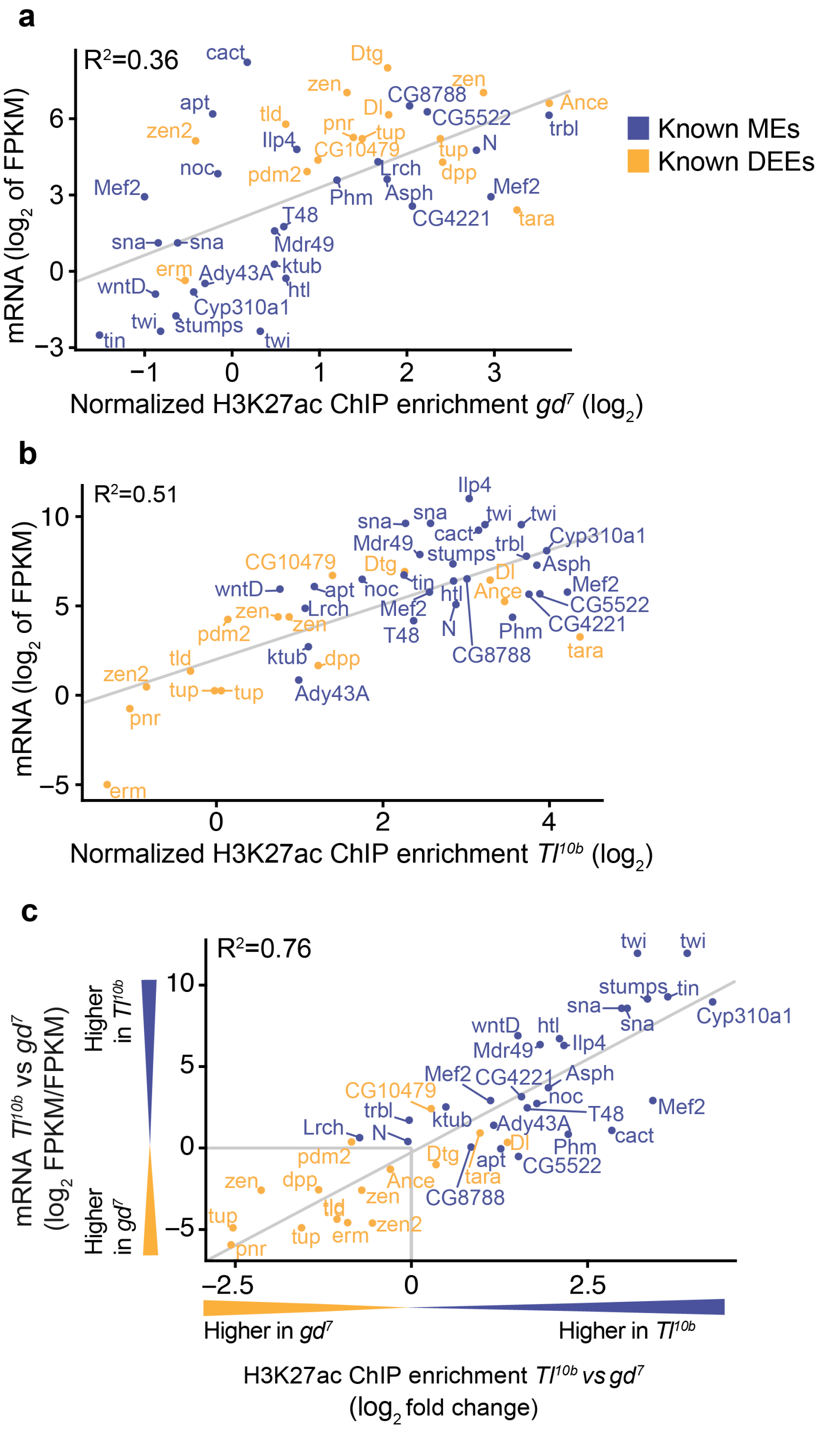

Fig. S1: Gene names for the data shown in Fig. 1a-c

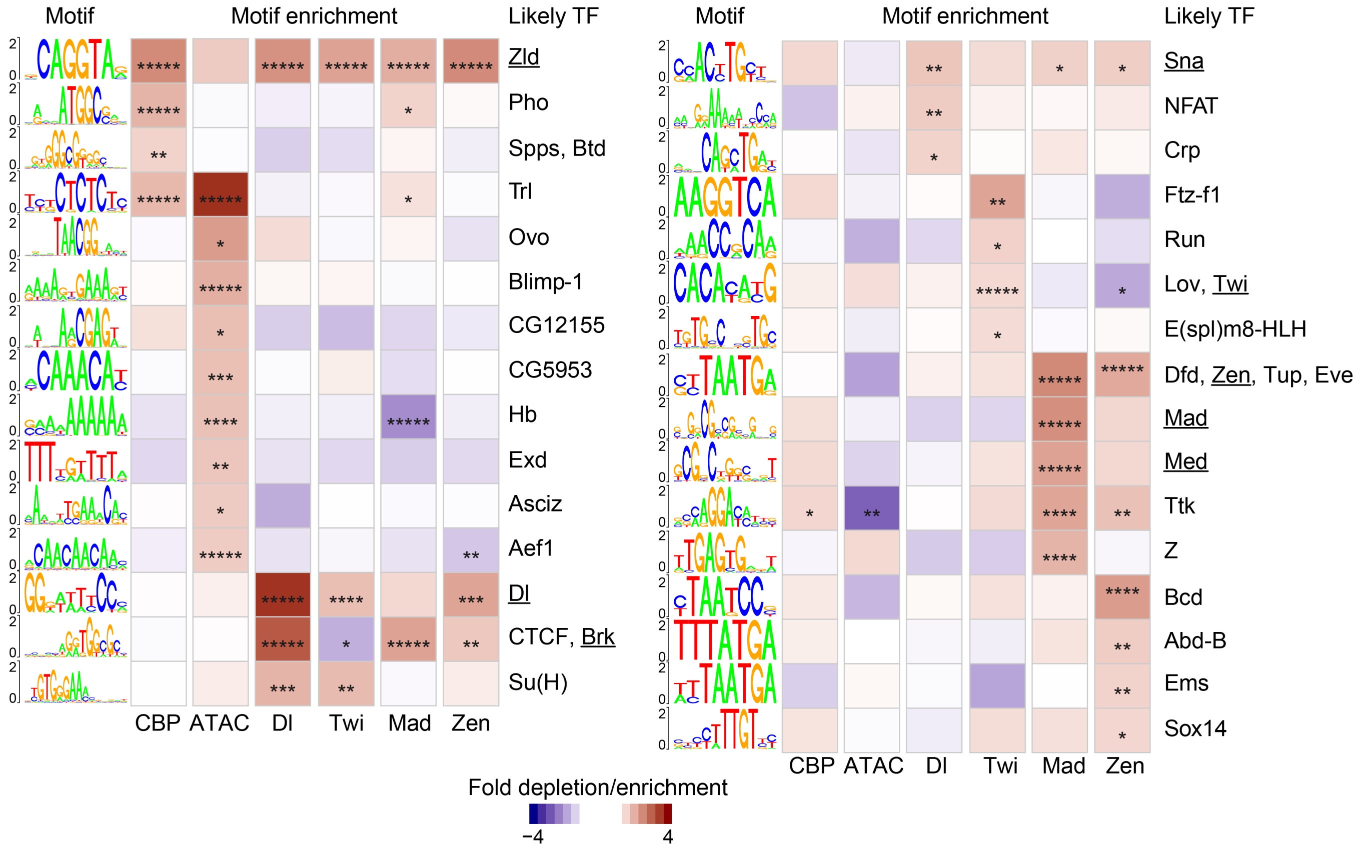

Fig. S2: Transcription factor motifs enriched in top ChIP-seq and ATAC-seq regions

Enrichment for known transcription factor motifs among the top 500 non-TSS regions among the CBP candidate regions, the ATAC-seq candidate regions, as well as the top regions bound by Dl, Twi, Mad or Zen, identifies DV transcription factor motifs but also many motifs that cannot be well explained and may not be tissue-specific. The top 500 non-TSS were compared to the remaining non-TSS peaks (see Methods). Note the lack of enrichment of known DV transcription factor motifs among CBP and ATAC-seq regions. However, a number of motifs enriched only in the top ATAC-seq regions are repetitive and/or display high AT content, suggesting a potential sequence bias in the assay. Dl, Twi ChIP-seq were performed in Tl^{10b} embryos, CBP ChIP-seq and ATAC-seq in wild-type embryos and Mad, Zen ChIP-seq in gd^7embryos. Significance was determined by a one-sided proportion test (* p <0.05, ** p < 10^{-2}, *** p < 10^{-3}, **** p < 10^{-4}, ***** p < 10^{-5}). Asciz is also known as CG14962 and Spps is also known as CG5669.

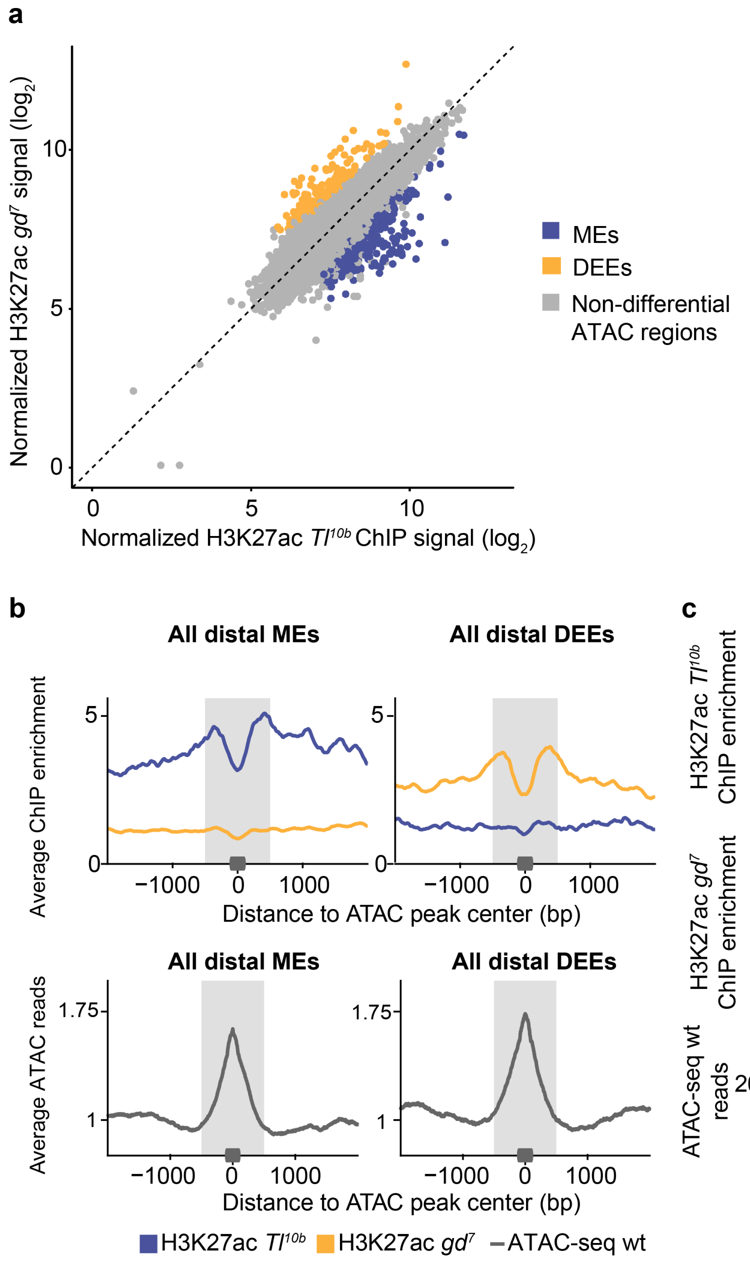

Fig. S3: Differential H3K27ac analysis of ATAC-seq regions is an effective method to identify tissue-specific enhancers

a *De novo* identification of DV enhancers based on differential H3K27ac. ATAC-seq data in wild-type embryos were used to locate a large number of putative enhancers. In 1000-bp windows centered on these ATAC-seq regions, average normalized H3K27ac signal among all replicates in gd^7 and Tl^{10b} embryos, respectively is shown as a scatterplot. Significant differences detected by DESeq2 are shown in blue (MEs) and yellow (DEEs). ATAC-seq regions not significantly different for H3K27ac between tissues are shown in grey and termed 'Non-differential ATAC regions'. **b** Average enrichment of H3K27ac over input in gd^7 and Tl^{10b} embryos, as well as

ATAC-seq signal from wild-type embryos (normalized to reads per million), are shown for MEs and DEEs that were located distally, at least 1 kb from any TSS. Note that the flanking nucleosomes with highest H3K27ac levels appear to be less sharp when compared to the CBP-derived regions shown in Fig. 1d. The light grey bar represents the 1 kb window used to calculate H3K27ac enrichments and the dark grey box represents the 200 bp enhancer region. **c** Examples of identified distal enhancers: zfh1-ME1 and C15-DEE2 (those were shown in Fig. 1e as CBP-derived enhancers). The light grey bar represents the 1 kb H3K27ac window and the blue and yellow boxes represent the 200 bp ME or DEE enhancer region, respectively.

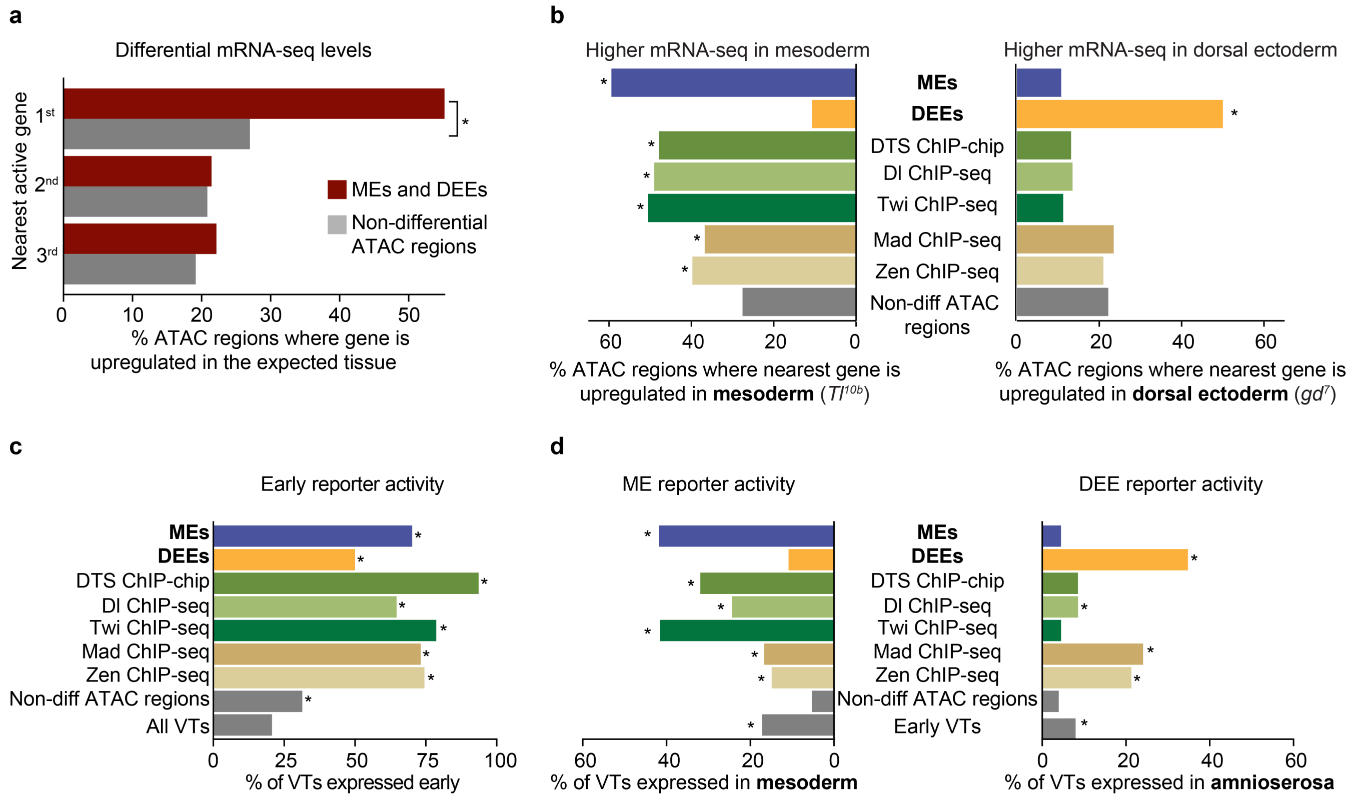

Fig. S4: Genes near putative ATAC-seq derived enhancers are differentially regulated across tissues

a As when using CBP, a large fraction of the active nearest genes near MEs and DEEs derived from ATAC-seq regions are differentially expressed in the expected tissue based on mRNA-seq data. Significance compared to non-differential ATAC control regions was determined using the one-sided chi-squared test ($p < 0.01$). The second and third nearest active genes near MEs and DEEs were not enriched over the control. **b** The closest active

genes near MEs and DEEs show differential gene expression in the expected tissue more frequently than the top 400 regions identified by transcription factor ChIP-seq. Significance over non-differential ATAC regions was determined using the Fisher's exact test ($p < 0.01$). **c** Transgenic reporter activity of Vienna Tiles (VTs) that overlap MEs, DEEs and transcription factor ChIP regions frequently show early embryonic expression (stages 4-10 in any tissue). Note that ATAC-seq derived regions do not perform as well as their corresponding CBP-derived regions shown in Fig. 2. Significance over all VTs was determined using the Fisher's exact test ($p < 0.01$). **d** VTs reporter expression that overlap MEs and DEEs is more tissue-specific than those overlapping transcription factor ChIP regions, although less than the CBP-derived regions shown in Fig. 2. Reporter expression was scored based on the annotations by Kvon et al. [1] as either "mesoderm" (left) or "amnioserosa" (right). Significance compared to non-differential ATAC regions were determined using Fisher's exact test ($p < 0.01$). Number of regions: all VTs (7705), early expressed VTs (1595), VTs overlapping putative ATAC-derived DV enhancers (112), MEs (66), DEEs (46). Dl, Twi ChIP-seq were performed in Tl^{10b} embryos and Mad, Zen ChIP-seq in gd^{7} embryos.

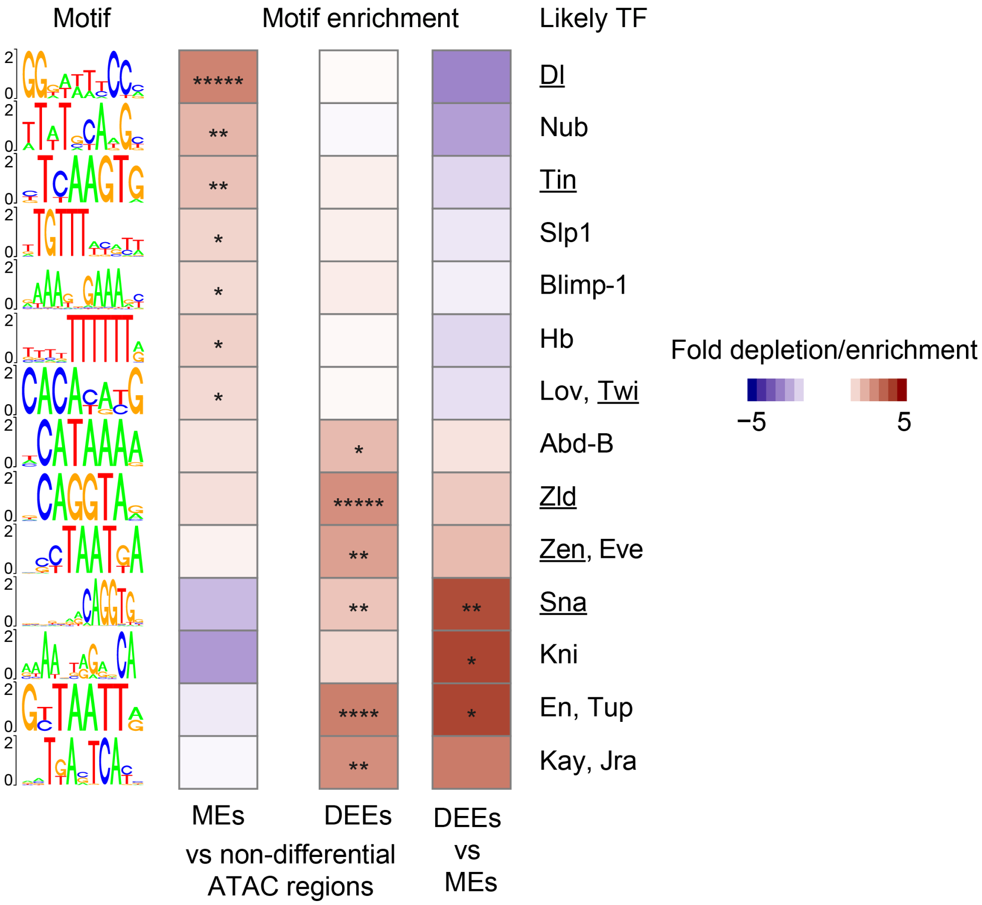

Fig. S5: Putative DV enhancer regions derived from ATAC-seq are enriched for known DV transcription factor motifs

Identification of transcription factor motifs (among all known *Drosophila* motifs) that are enriched at putative MEs and DEEs derived from ATAC-seq regions over non-differential ATAC control regions are shown as sequence logos in bits. In the case that several transcription factors are known to bind to the same motif, all matches are shown on the right and factors that are known to function in DV patterning are underlined. Nearly all DV transcription factors discovered via CBP-derived regions and shown in Fig. 3a are also recovered in the ATAC-seq derived regions. Significance was determined by a one-sided proportion test (* $p < 0.05$, ** $p < 10^{-2}$, *** $p < 10^{-3}$, **** $p < 10^{-4}$, ***** $p < 10^{-5}$).

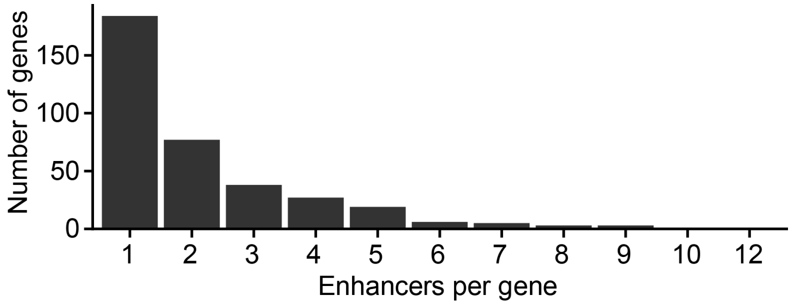

Fig. S6: Number of genes with one or multiple assigned enhancers

51% of genes (186 of 365 total genes) have only one assigned DV enhancer, while the remaining genes have multiple (up to 12).

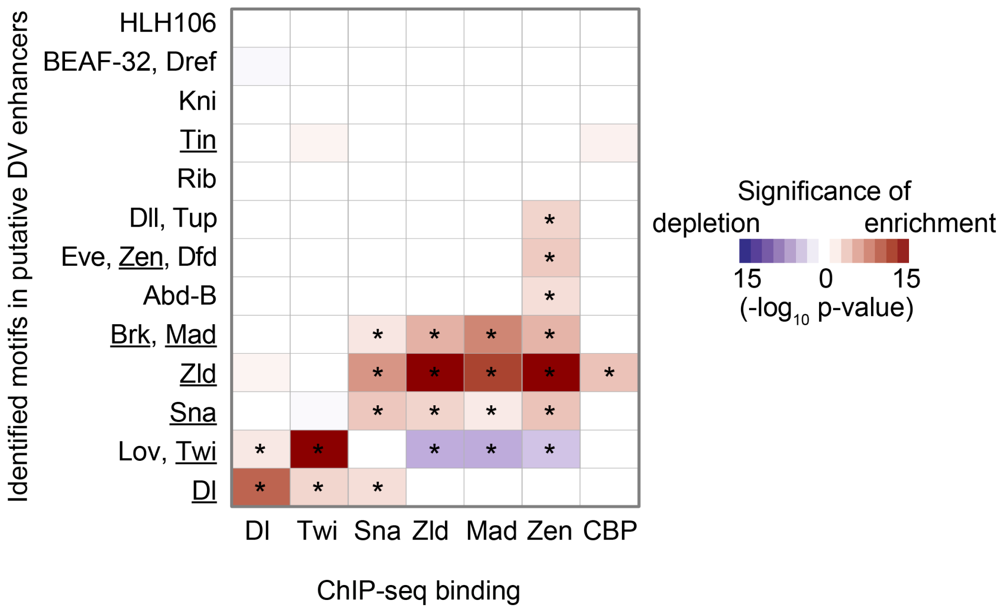

Fig. S7: Transcription factor ChIP-seq signal is preferentially found at expected binding motifs present within putative DV enhancers

Transcription factor ChIP-seq enrichment values for Dl, Twi, Sna, Zld, Mad, Zen and CBP at all MEs and DEEs that have the respective motif were compared to the ChIP-seq enrichment values at MEs and DEEs without the respective motif. Significant enrichment or depletion was determined by a two-sided Wilcoxon test and marked with a star ($p < 0.05$).

Supplemental figure references

1. Kvon EZ, Kazmar T, Stampfel G, Yanez-Cuna JO, Pagani M, Schernhuber K, et al. Genome-scale functional characterization of Drosophila developmental enhancers in vivo. Nature. 2014.
